# Dynamical Nonequilibrium Molecular Dynamics Simulations Reveal Atomistic Steps of C‑Type Inactivation in Cardiac hERG Channels

**DOI:** 10.1021/jacs.6c05939

**Published:** 2026-08-25

**Authors:** Flavio Costa, Carlos Bassetto, Francisco Bezanilla, Alberto Giacomello

**Affiliations:** † Dipartimento di Ingegneria Meccanica e Aerospaziale, 9311 Sapienza Università di Roma, Rome 00184, Italy; ‡ Department of Physics and Astronomy, 12346 UT San Antonio, San Antonio, Texas 78249, United States; § Department of Biochemistry and Molecular Biology, 28068 The University of Chicago, Chicago, Illinois 60637, United States; ∥ Institute for Biophysical Dynamics, The University of Chicago, Chicago, Illinois 60637, United States; ⊥ Centro Interdisciplinario de Neurociencias, Facultad de Ciencias, Universidad de Valparaiso, Valparaiso 2284440, Chile; # Facultad de Ciencias del Mar y Recursos Naturales, Universidad de Valparaíso, Valparaiso 2284440, Chile

## Abstract

hERG is a voltage-gated potassium channel whose malfunction is associated with cardiac pathologies. Unlike other potassium channels, hERG exhibits a peculiarly fast C-type inactivation at the level of the selectivity filter (SF), which has complicated our understanding of its structural dynamics. Molecular Dynamics (MD) simulations offer powerful tools to probe ion channel gating, but conventional equilibrium MD simulations are often unable to capture fast transitions. Despite their potential, Dynamical Nonequilibrium MD simulations (D-NEMD) have rarely been applied to understand ion channel mechanisms. Here, we combine equilibrium MD simulations, D-NEMD simulations, and electrophysiology assays to atomistically characterize a possible pathway of hERG C-type inactivation. First, we show that the ion occupancy within the SF influences hERG propensity to spontaneously inactivate. Then, we reveal three possible major steps that underlie hERG inactivation: (i) the disruption of contacts between residues of the SF, P-helix, and S5–P helix, (ii) the flipping of V625, which ultimately (iii) constricts the SF, leading to the final nonconductive state of the channel. Mutagenesis and electrophysiological recordings confirm the functional relevance of the computationally identified residues. Overall, our results demonstrate the utility of D-NEMD in investigating rapid nonequilibrium transitions in physiologically relevant complex proteins, such as ion channels, which remain a challenge for conventional equilibrium simulations and structural methods, such as X-ray crystallography and cryo-EM.

## Graphical abstract

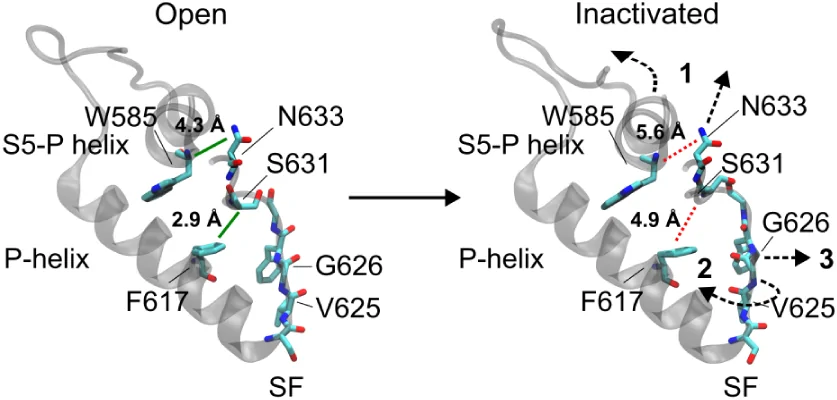

## Introduction

The human ether-à-go-go-related gene (hERG or *KCNH2*) encodes a voltage-gated potassium channel (K_v_ channel) expressed in cardiac cells that plays a vital role in the repolarization phase of the cardiac action potential.^1–3^ It consists of four identical subunits, each composed of six transmembrane segments (S1–S6), organized into two functional domains^4^ (Figure 1A): the Voltage Sensor Domain (VSD), encompassing segments S1–S4, which senses membrane potential variations and triggers channel opening; the Pore Domain (PD), comprising S5, S6, the P-helix, and the Selectivity Filter (SF), physically controls channel opening and closing. hERG is an important pharmacological target, and its malfunctions are associated with severe cardiac pathologies, including long QT syndrome type 2 (LQTS-2) and short QT syndrome, which can promote arrhythmias and sudden death.^5^

**Figure 1:**
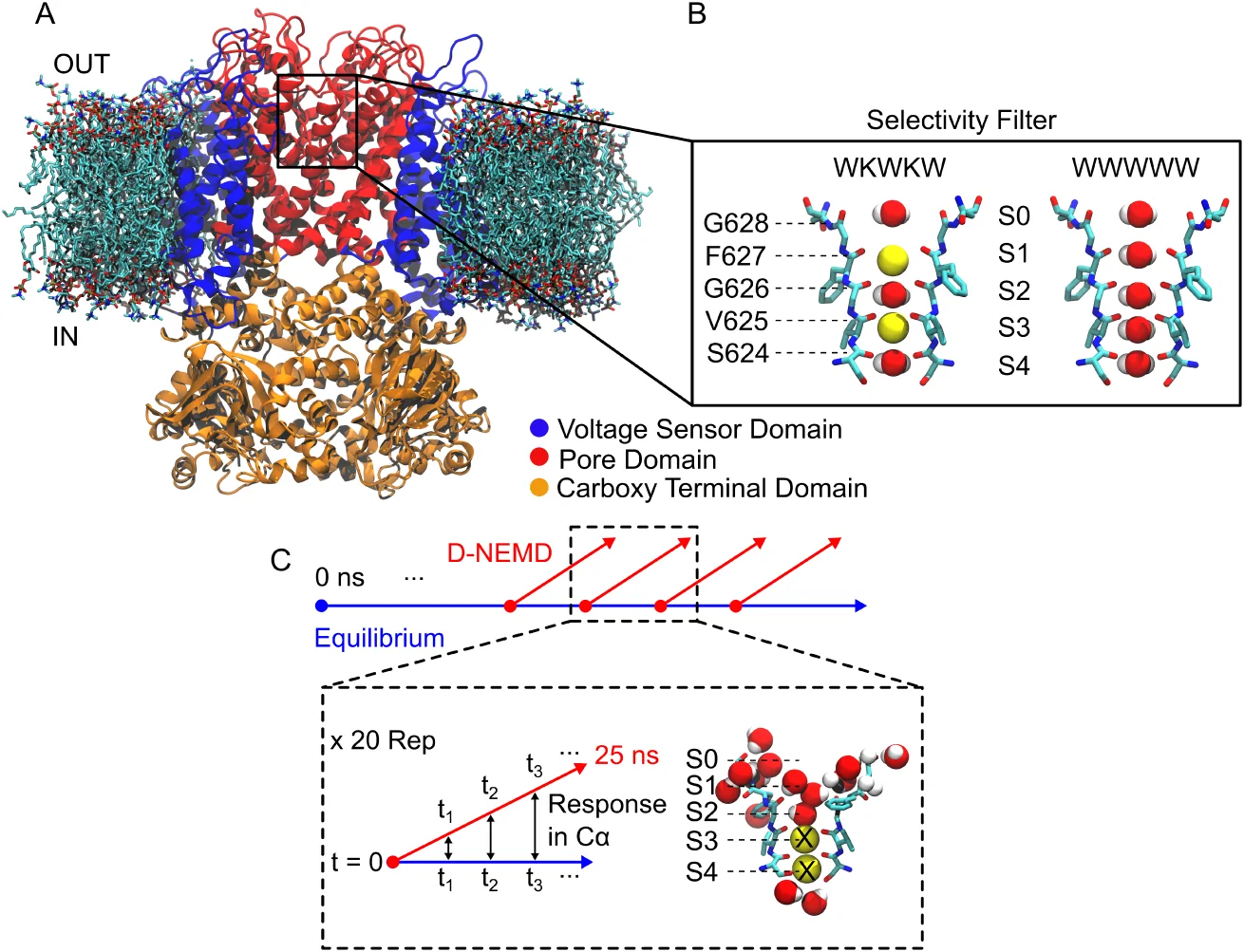
Computational model and protocol used to study the hERG channel. (A) hERG channel embedded in a POPC lipid bilayer; the Voltage-Sensor Domain is shown in blue, the Pore Domain in red, and the Carboxy-terminal Domain in orange. (B) SF configurations used to start the equilibrium simulations. K^+^ ions are colored yellow and displayed in vdW representation, like the water molecules. The residues of the SF and the corresponding occupancy sites (S0 to S4) are reported. (C) Scheme of the procedure used to set up the D-NEMD simulations. From equilibrium simulations of the systems started with 2 K^+^ at 0 mV with the CHARMM36 force field, 20 independent simulations were performed, applying the removal of the K^+^ ions from the SF as a perturbation. For each D-NEMD replica, equivalent time points were combined to measure the C_α_ deviation of the protein amino acids from their equilibrium.

hERG channels can cycle through three functional states: closed, open, and inactivated. The inactivation process, the open-to-inactivated transition, describes an ion channel entering a nonconducting state even though an activating stimulus is still present. The rate of this transition varies among different ion channel families,^6^ which contributes to their specific physiological functions. hERG exhibits an exquisitely fast inactivation occurring at the PD level, i.e., so-called C-type inactivation, with kinetics in the millisecond range,^7^ substantially faster than other K_v_ channels.^8^ These rapid kinetics, combined with voltage dependence and K^+^ sensitivity,^9,10^ have made the structural characterization of inactivation challenging. Computational studies have suggested that hERG inactivation is characterized by an SF constriction, with distances between G626 residues of opposing subunits less than 5 Å ^11,12^ and/or by an extracellular dilation of 3 Å.^13^ Recently, Lau et al.^14^ experimentally solved a hERG inactivated structure, revealing that the rotation of the V625 side chain from the center of the SF toward S620 represents a key determinant of hERG C-type inactivation. Although some characteristics of the SF in the inactivated state have been highlighted, the atomistic sequence and dynamics of structural conformational events leading from the open to the inactivated state remain largely unclear.

Molecular Dynamics (MD) simulations are making significant strides toward a better approximation of physiologically relevant processes.^15^ In the field of ion channels,^16^ the synergy between experiments and simulations is fueled by the increasing availability of structures and high-quality electrophysiological data. However, progress is mostly related to simulations of equilibrium^17^ or stationary^18^ conditions, where extracting signals from the prevalent thermal noise can be improved by larger computational effort or quasi-static approximations.^19^ Truly nonequilibrium processes in which a time-varying response is triggered by an external stimulus are ubiquitous in physiology but remain challenging to tackle in molecular simulations. To overcome these limitations, we employ Dynamical Nonequilibrium MD (D-NEMD) simulations, which offer a powerful method for probing the response of proteins to perturbations and elucidating how they rearrange after such events.^20^ Several systems have been studied employing D-NEMD simulations, spanning from CFTR channels^21^ to SARS-CoV-2 ^22,23^ and DNA gyrase.^24^ In this approach, starting from equilibrated systems obtained via equilibrium MD simulations, multiple short nonequilibrium simulations are run in which a perturbation, typically the removal of a ligand, is introduced to push the system out of equilibrium. The Kubo–Onsager relation^20,25^ is used to measure the linear response of the protein to this perturbation while the system relaxes back to equilibrium. Thus, D-NEMD simulations represent an invaluable computational approach to study biological processes that are out of equilibrium and characterized by fast kinetics, such as hERG C-type inactivation. Nevertheless, despite their appeal, this approach has been scarcely applied to understanding ion channel function.

In this work, we investigate the atomistic pathway of hERG C-type inactivation using a combination of computational (equilibrium and nonequilibrium MD simulations) and experimental (electrophysiology) assays. First, we show that when starting hERG in the open state without K^+^ ions inside the SF, the channel spontaneously inactivates. The CHARMM36 force field tends to facilitate this process compared to AMBER. Then, using the removal of K^+^ ions from the SF as a perturbation in D-NEMD simulations, we map the sequence of structural rearrangements that drive the inactivation. Using a voltage step as a perturbation provides analogous results, confirming the robustness of our findings. Lastly, we predict the relevant interactions that are broken during the inactivation process, confirming the role of the involved residues through site-directed mutagenesis and electrophysiological recordings. Overall, our results suggest three major steps of hERG C-type inactivation: (i) disruption of interactions among the SF, P-helix, and S5–P helix, which promotes structural rearrangements in the SF; (ii) rotation of V625, which leads to (iii) subsequent constriction of the filter. By resolving the finely tuned dynamics of hERG C-type inactivation, this approach provides a robust basis for investigating the molecular mechanisms underlying both inherited and drug-induced channelopathies. Moreover, our work demonstrates that D-NEMD simulations represent a powerful tool for capturing atomistic details of rapid transitions in complex biomolecules as well as for exploring transient states of ion channels.

## Results

### Equilibrium MD Simulations of the hERG Open State

To investigate the molecular determinants of hERG C-type inactivation, the hERG open state (PDB: 5VA2)^26^ was studied via equilibrium MD simulations. The selectivity filter (SF) was initialized in the WKWKW configuration (Figure 1A–B), with the S1/S3 sites occupied by potassium ions (K) and S0/S2/S4 by water molecules (W). This arrangement corresponds to the soft knock-on permeation mechanism of potassium channels^27–29^ and has been previously used in hERG simulations.^11^ During the equilibration, this state evolved rapidly into a WWWKK configuration^11,30^ —hereafter named as “2 K^+^” (Figure S1A). C-type inactivation was monitored via the rotation of V625 and SF constriction, which are considered hallmarks of the inactivated state.^11,12,14^ During 500-ns simulations, no events of V625 flipping or SF constriction were observed, suggesting that the system maintained an open, conductive configuration of the filter (Figure S1B–C).

Both simulations^11^ and experiments^14,31,32^ showed that the occupancy of the SF plays a major role in inactivation. For this reason, a further set of equilibrium simulations (see *Methods*) was performed with different starting SF configurations, force fields, and voltages. The previously identified stable WWWKK configuration (2 K^+^) was studied together with the WWWWW configuration (0 K^+^; Figure 1B), in which the filter was devoid of ions. Different voltages (V_*z*_ = 0 and +900 mV) were chosen to mimic the membrane potential variation occurring during inactivation, using both the AMBER and CHARMM36 force fields, which are known to differently capture ion permeation and inactivation of ion channels.^33,34^ For both force fields, the 2 K^+^ systems at 0 mV maintained their initial WWWKK configuration for most of the time (Figure S2A–B).^11^ AMBER 0 K^+^ systems at 0 mV showed an ion entering from the intracellular side of the SF which preferentially occupied the S3 or S4 sites (Figure S2C), while, in CHARMM36 0 K^+^ systems at 0 mV, ions intermittently entered the filter from the extracellular side, reaching up to the S2 and S3 sites (Figure S2D). At +900 mV, the ion initially present in the filter of the AMBER 2 K^+^ system at position S3 left the filter, although, in replicas, 2 different ions occupied S3 during the entire simulation (Figure S2E). Finally, in the CHARMM36 2 K^+^ system at +900 mV, a general translocation of the ions toward the top side of the SF was revealed (Figure S2F). Overall, these results indicate that SF ion occupancy and permeation paths depend on both the force field and the applied voltage, while remaining consistent with a conductive SF at 0 mV.

The structural modifications on the SF were also monitored. With the AMBER force field, the 2 K^+^ systems showed no V625 rotation during 1.4-μs simulations, thus remaining in the open state (Figure S3A), in accordance with previous evidence.^14^ In contrast, removing ions from the SF, both at the beginning of the simulations or under high voltage (V_*z*_ = +900 mV), induced the rotation of at least three V625 residues in replica 3 (Figure S3B) and replicas 1 and 3 (Figure S3C), respectively. Similarly, CHARMM36 simulations showed no V625 rotation in 2 K^+^ systems (Figure S3D), which was instead observed in three subunits for 0 K^+^ or under +900 mV (Figure S3E–F). The G626-G626 distance followed this pattern: in AMBER 2 K^+^ systems, it remained >5 Å, consistent with an open, conducting state (Figure S4A).^26^ Most 0 K^+^ replicas also retained a conductive filter (Figure S4B), while the voltage caused collapse in two of the three replicas (Figure S4C). CHARMM36 2 K^+^ systems remained open (Figure S4D), but both 0 K^+^ and V_*z*_ = +900 mV systems showed asymmetric constriction (Figure S4E–F). Finally, the bending angle of helix S6 (θ), delimiting the pore, was analyzed. In both AMBER and CHARMM36 systems initiated with two K^+^ ions in the SF, the pore predominantly retained an open/inactivated conformation, although some subunits displayed θ values closer to the closed-state conformation observed in EAG1 (Figure S5A–B).^35^ In simulations without ions in the SF, the pore generally remained open/inactivated, except for CHARMM36 replica 1, where three subunits adopted conformations consistent with a putative closed state (Figure S5C–D). Under +900 mV, AMBER systems maintained an open/inactivated pore throughout the simulations, whereas CHARMM36 systems exhibited intermediate θ values between the open/inactivated and closed conformations. No direct correlation was found between the bending of helix S6 and V625 flipping, filter constriction, or ion permeation events (Figures S2–S4), indicating that the structural rearrangements of the filter are decoupled from those occurring in the pore. However, such events occur when the pore is in the open/inactivated configuration, in agreement with previous works.^36–40^ Since the V625 rotation in three subunits has been suggested to be sufficient for inactivation,^14^ all these findings together demonstrate: (i) the ability of the filter to spontaneously inactivate when it is started without ions; (ii) a greater propensity of CHARMM36 over the AMBER force field to produce the inactivated state of the channel in the simulated time, in agreement with the literature;^13,34,41^ (iii) an analogy between the inactivating effect of depolarization and the artificial removal of K^+^ ions from SF.

To validate the inactivated structures obtained during the equilibrium simulations initiated from the open state (PDB: 5VA2), cluster analysis was performed, considering only trajectories in which the channel transitioned into the inactivated state (see *Methods*; Figure S6A). Representative SF conformations were then compared with the experimental low-K^+^ cryo-EM structure^14^ (PDB: 9CHQ, Figure S6B) which corresponds to the hERG inactivated state. All inactivated conformations showed an RMSD <1.5 Å relative to the experimental low-K^+^ structure, confirming the accurate reproduction of the inactivated state. Finally, comparison of the SF radius profiles revealed that the CHARMM36 simulations with 0 K^+^ ions in the SF at 0 mV best reproduced the radius profile of the experimental inactivated-state structure (Figure S6C)—see, for example, at the level of V625. Overall, both K^+^ removal and high voltage effectively reproduced the inactivated state of hERG in simulations, with the CHARMM36 force field and an initially ion-depleted SF showing the greatest tendency to inactivate it from the open state.

### Dynamical Nonequilibrium MD Simulations to Reproduce hERG C-Type Inactivation

Inactivation is a conformational change due to a variation in the membrane potential and, as such, is a nonequilibrium process. Due to the rapid dynamics of hERG C-type inactivation,^4,26^ Dynamical Nonequilibrium MD simulations (D-NEMD)^21^ are well suited to capture how the SF structurally rearranges in time toward the inactivated state. The two crucial ingredients of D-NEMD are the initial state and the perturbation.^42^ On the basis of the results of the previous section, the CHARMM36 force field was selected due to its faster inactivation (Figures S3 and S4), and the initial state was chosen as the equilibrated open state in the WWWKK configuration of the SF (Figure 1B). D-NEMD simulations were performed by running 20 independent replicas of 25 ns each at 303.15 K. Building on the evidence that the ion occupancy of the SF plays a key role in inducing C-type inactivation, the chosen perturbation was the instantaneous removal of K^+^ ions from the SF, expected to bring the system out of equilibrium, creating a driving force that tends to relax the channel toward a new equilibrium state (Figure 1C), the inactivated one. In short, the ion removal from the SF using the CHARMM36 force field was used as a computationally convenient and physically consistent way of simulating inactivation induced by depolarization, avoiding the use of very high and nonphysiological voltages that may introduce artefacts on the protein structure.

Using the Kubo–Onsager relation,^20,25^ the flipping of V625 and the SF constriction were computed by averaging the 20 independent 25-ns D-NEMD simulations. These events occurred 2.5 and 3.5 ns after the perturbation (Figure S7), respectively, indicating that the transition from the open to the inactivated state was completed within 4 ns after applying the perturbation. Interestingly, the rotation of V625 started before SF constriction, confirming the hypothesis that the constriction results from filter rearrangement triggered by V625 flipping.^14^

The C_α_ displacement of the protein amino acid residues and the contacts between key residues were subsequently analyzed to dissect the atomistic details of the structural rearrangement of the SF after the perturbation. Again, the Kubo–Onsager relation^20,25^ was used. The C_α_ displacement showed that the sequences of residues mostly affected by K^+^ ion removal are Q576 to D580 and N598 to L602. These segments are localized on the flexible loops between the P-helix and the SF (Figure 2A, Table S1) which have been shown to influence activation and inactivation.^11,14,32,43^ In the SF, G626 and G628 were characterized by the greatest displacements (after 5 ns, C_α_ displacement >2 Å). Notably, S631, which is known to affect inactivation when mutated,^31,44^ had a C_α_ response of 2.6 Å, the highest value among the residues on the loop between the SF and helix S6 (Table S1). At 25 ns, statistically significant directional displacements (assessed as |mean| > standard deviation) were identified within the N573–T634 segment (Table S2). The S5–P helix (residues W585–I593) exhibited the most coherent signal: all residues showed a significant negative Δy (−0.56 to −1.91 Å), indicative of a rigid-body-like displacement along the negative *y*-axis, accompanied by a significant positive Δz in W585–L589 and D591 (+0.48 to +0.82 Å), and a significant positive Δx emerging progressively in the C-terminal portion (residues G590–I593; + 0.83 to +1.18 Å). This spatial gradient across the helix is consistent with a tilting motion around an oblique axis. The P-helix (residues K608–T623) displayed a dominant positive Δx displacement in 10 out of 16 residues (+0.34 to +1.09 Å, all positive), concentrated in the N-terminal half (K608–F617), with the signal attenuating toward the C-terminus; a modest positive Δy was significant in K608, K610–Y611, A614–L615, F617, and a positive Δz was significant in 10 residues, suggesting a predominantly translational movement along x with a minor torsional component. The SF showed a highly specific axial signal, with 4 out of 5 residues exhibiting significant positive Δz displacements (+0.68 to +1.06 Å) and no significant Δx or Δy, consistent with a net displacement along the pore axis. Finally, the C-terminal loop (residues N629–T634) underwent a concerted three-dimensional displacement, with all residues significant along Δx (+0.95 to +1.43 Å) and Δy (+0.61 to +1.33 Å), and five out of six along Δz (+0.75 to +1.92 Å), all positive. Collectively, these results describe a coordinated conformational rearrangement in which the S5–P helix tilts in the −y/+z direction, the P-helix translates along +x, and the SF shifts axially along +z (Movie S1–S2).

**Figure 2:**
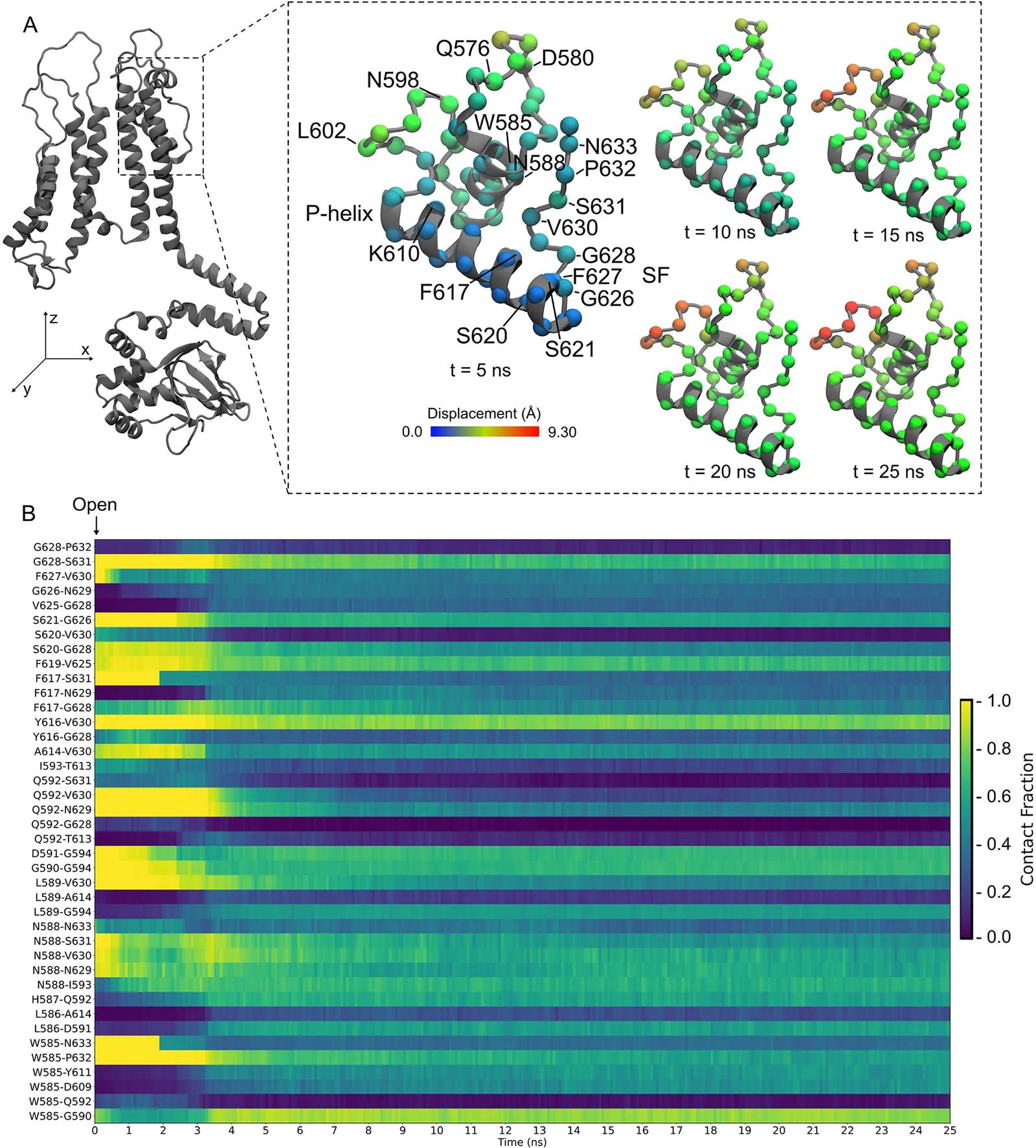
Structural response of hERG to the removal of K^+^ ions from the SF. (A) C_α_ displacement of the protein’s amino acids computed for each residue following K^+^ ions removal from the SF. Ensemble averages are taken for each of the shown instants over the 20 D-NEMD replicas and over the four protein subunits. (B) Contact map showing intrasubunit interactions computed from D-NEMD simulations, where a contact fraction equal to 1 corresponds to the presence of the interaction between pairs of residues, while equal to 0 corresponds to its complete absence.

Figure 2B and Movie S3 describe the time evolution of contacts (see *Methods* for the definition of contact) between residues in the selectivity filter (SF), the P-helix, and the S5–P helix (between helix S5 and the P-helix), highlighting the formation, persistence, and disruption of key interactions during the simulations. Several contacts, G628–S631, S621–G626, and Y616–V630, remain stable throughout the simulations, with contact fractions (CF) > 0.7, indicating structural conservation. A subset of interactions shows early destabilization or clear time-dependent transitions. G590–G594 and D591–G594 are conserved within the first 1.5 ns (CF > 0.9) and subsequently break (CF ≈ 0.4) for the remainder of the simulation. F617–S631 exhibits a high initial interaction (CF ≈ 0.9) followed by a complete loss of contact after ∼2.0 ns (CF < 0.2), indicating a pronounced local conformational rearrangement early in the process. W585–N633 is stable during the first ∼2.0 ns (CF > 0.9), then decreases in frequency (CF ≈ 0.5) and finally breaks after ∼3.2 ns (CF < 0.3). Some interactions emerge or become stabilized during later stages of the simulation. W585–G590 is absent during the first ∼3.2 ns (CF ≈ 0.3), then forms and remains conserved for the rest of the trajectory (CF > 0.8). L586–D591 is absent in the open state (CF ≈ 0.2) but increases in occurrence after ∼3.3 ns, reaching CF > 0.6. N588–V630 is initially present, is disrupted at ∼0.8 ns, briefly reappears (CF ≈ 0.5 for ∼2 ns), reemerges again at ∼3.2 ns, and finally breaks after ∼3.8 ns. L589–V630 remains largely unchanged over time but shows a tendency to weaken after ∼4 ns. F617–G628 shows no clear interaction within the first ∼2.0 ns, followed by a moderate increase (CF ≈ 0.6 around ∼3.5 ns) with a slight decrease thereafter. W585–P632 exhibits a single transition at ∼5.0 ns, where an initially stable contact (CF > 0.8) is lost (CF < 0.4). Overall, these results highlight a dynamically reorganizing interaction network involving the SF, P-helix, and S5–P helix, where coordinated formation and disruption of contacts accompany the conformational transition associated with inactivation.

To check the robustness of the identified pathway, 4 independent replicas of D-NEMD simulations were also run for 150 ns each at 303.15 K, starting from the WWWKK configuration of the SF and using a voltage of +900 mV as a perturbation. The reason behind the choice of such a large voltage was based on the evidence that lower voltages, +500 mV and +700 mV, did not trigger any ion movements during the 500-ns equilibrium simulations with AMBER force field, which were previously suggested to be determinant to induce the filter inactivation, thus showing the need for a higher voltage to perturb the system within computationally accessible times (Figure S8). At +900 mV, the structural rearrangements of V625 and G626 occurred after 75 ns (Figure S9A). Consistent with the ion-removal perturbation, the residues characterized by the greatest C_α_ displacement were localized on the flexible loops between the P-helix and helix S5, and the SF and helix S6 (Figure S9B, Table S3) as well as G626, G628, and S631. Interestingly, the contact formation and breaking between residues of the SF, P-helix, and S5–P helix resemble the pattern found in the D-NEMD simulations with the removal of K^+^ ions from the SF as a perturbation (Movie S4). However, the most relevant events, i.e., the formation and breaking of interactions that are present only in the open or in the inactivated state, such as F617–S631, occurred in the first 10 ns, thus being uncoupled from the V625 flipping and the filter constriction (Figure S9C). The directional displacement analysis (Table S4) revealed that the P-helix and N629–T634 behaved consistently with the D-NEMD simulations with K^+^-removal: the P-helix retained a significant positive Δx in its N-terminal half (+0.55 to +1.38 Å), and N629–T634 showed uniform positive Δx and Δy. In contrast, the P-helix developed a dominant positive Δz signal (+0.29 to +1.06 Å) absent in the D-NEMD simulations at 0 mV, indicating that sustained voltage drives a superimposed axial displacement toward the pore axis. The most striking difference was observed at the S5–P helix: the significant negative Δy characterizing all residues in the 25-ns D-NEMD with K^+^-removal was absent under +900 mV, replaced by a progressive positive Δx (+1.07 to +1.83 Å). The SF showed a convergent positive Δz in agreement with D-NEMD with K^+^-removal, but under depolarization this signal was accompanied by a significant negative Δy at residues S624–V625 and by markedly reduced inter-replica variance.

Overall, D-NEMD simulations performed with K^+^ ion removal and a large depolarizing voltage step as perturbations yielded consistent results, leading to similar inactivated structures that display hydrogen-bond networks comparable to those reported in Lau et al., 2024 ^14^ (Figure S10). The only exception was the Y616–N629 interaction, whose distance frequently exceeds the typical hydrogen-bond range but is still observed to occur through water-mediated interactions. The need for longer simulations using the depolarizing voltage step leads to higher computational costs, suggesting that K^+^ ion removal from the SF is a computationally convenient perturbation for inducing inactivation. We remark that there is a significant difference in time scales between the physiological millisecond inactivation kinetics and the nanosecond one observable in our D-NEMD simulations; comparisons of different force fields and perturbations show that this difference is due to the faster inactivating tendency of CHARMM36 and to the abrupt ion removal and high depolarizing electric potential used in the simulations. However, the consistent results across force fields, equilibrium configurations of the SF, and D-NEMD simulations with different perturbations suggest that the main sequence of events and residues involved is robust. The Cα displacement analysis during the D-NEMD simulations identified key domain motions associated with inactivation. Although this computationally efficient approach is likely to reliably capture major conformational changes, it provides only an indirect indication of which residues may play important functional roles and may overlook isolated side-chain movements. To address this limitation, we complemented the Cα displacement analysis with a more specific contact analysis focused on side-chain motions, revealing a dynamic pathway by which hERG undergoes C-type inactivation. This transition is characterized by three major steps (Figure 3): (i) the breaking of relevant interactions involving residues on the SF, P-helix, and S5–P helix is the starting point of a cascade of events that (ii) triggers the flipping of V625 and (iii) culminates in the filter constriction at the level of G626. These results agree with previous models,^32,45^ clarifying the temporal sequence of conformational changes underlying SF gating.

**Figure 3:**
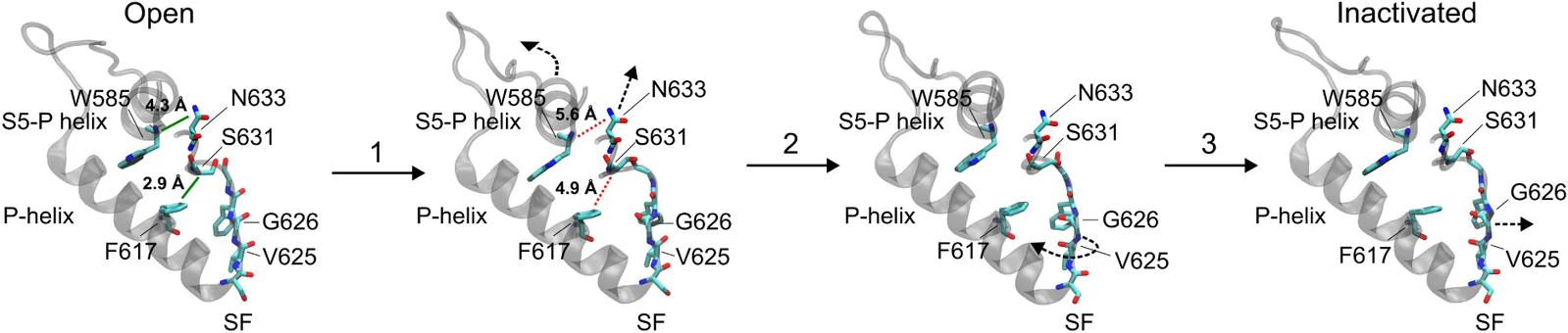
Proposed three-step-based pathway for hERG C-type inactivation. The first step involves the breaking of the N633–W585 and S631–F617 contacts. The second step involves the rotation of V625 in the SF, which allows/leads to the constriction of the filter at the level of G626 (third step). For simplicity, only one subunit is shown.

### Experimental Assays Confirm the Role of Key Residues on the SF, S5–P Helix, and P-Helix in hERG C-Type Inactivation

The computational analysis revealed the presence of interactions between the SF, the S5–P helix, and the P-helix, important for hERG C-type inactivation. Among them, F617–S631 and W585–N633 showed an evident rearrangement during the open-to-inactivated transition: the hydrophobic interaction between the bulky aromatic side chain of F617 and the backbone of S631, and the hydrogen bond between the nitrogen of the W585 backbone and the N633 side chain (Figure S11) were lost during the transition. These residues also showed the greatest C_α_ displacement values (Tables S1–S3)—excluding the residues on the flexible loops—and the highest difference in terms of contact fraction between the open and inactivated states (Figure 2 and Figure S9). In previous works, mutations of F617 and S631 have been shown to perturb the inactivation process in hERG channels, as well as the role of the SF and S5–P helix,^32,43,44,46,47^ thus supporting our computational predictions. Here, the role of W585 and N633 in hERG inactivation was further investigated using alanine mutations and electrophysiological assays. When either W585 or N633 were mutated to Ala, the channels exhibited no inactivation in a 10 s time frame, in sharp contrast with the WT (Figure 4A–D and Figure S12A–C). This result demonstrates the importance of these residues in the inactivation process whereby Ala mutations in those positions prevented hERG from entering the inactivated state.

**Figure 4:**
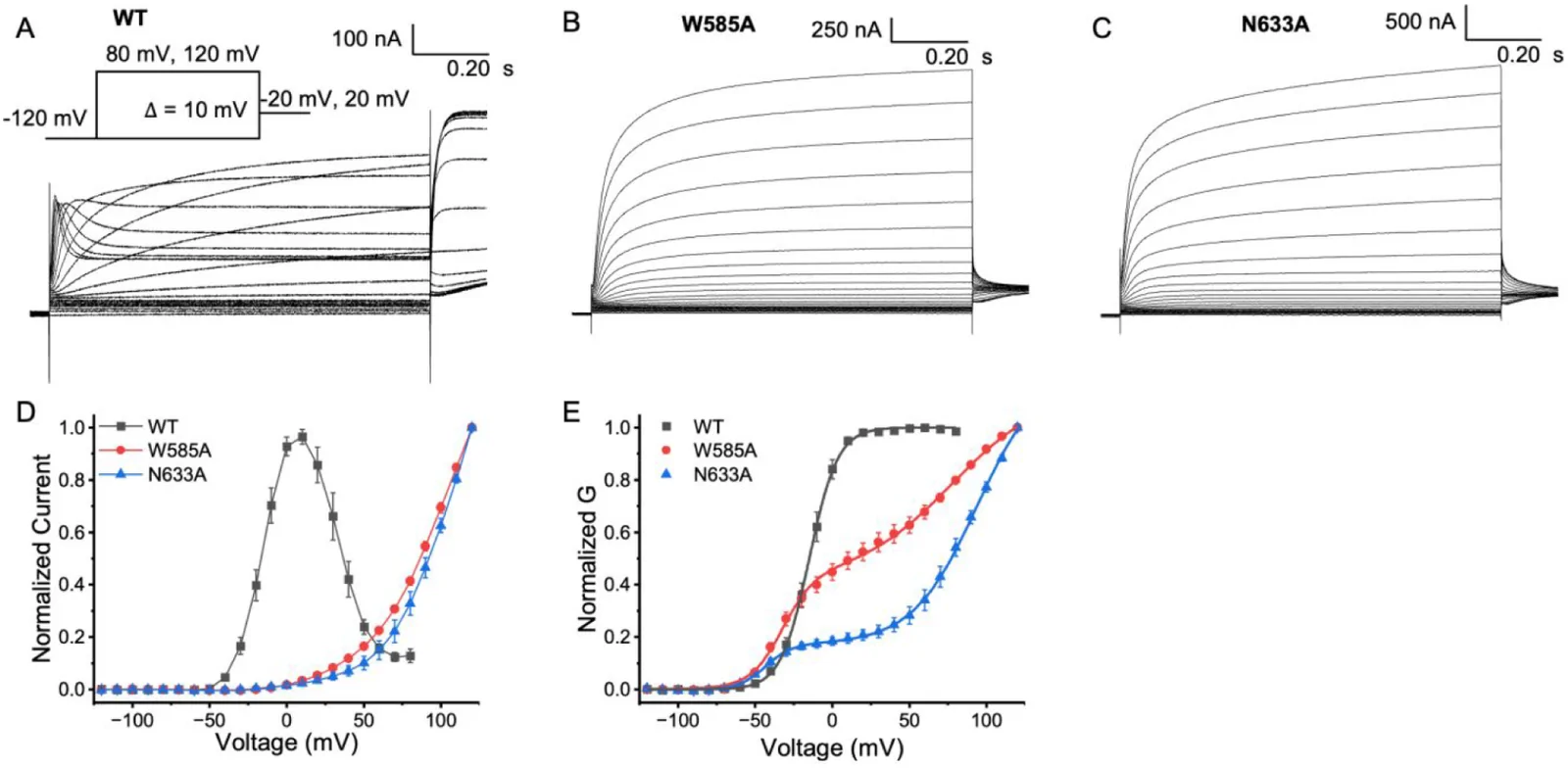
Alanine mutations at W585 and N633 abolish C-type inactivation in hERG channels. Current traces for WT (A), W585A (B), and N633A (C), respectively. Inset in (A) shows the voltage protocol used; because W585A and N633A are right-shifted and display no inactivation, the related protocols were slightly different from WT. For WT, the voltage pulses reached 80 mV, and for the mutants, 120 mV. The hyperpolarizing returning pulse was −20 mV for WT and 20 mV for the mutants. (D) The peak current normalized by its maximum, plotted against the membrane voltage. The current shown in (D) was corrected for the leak. The raw normalized current without leak subtraction is shown in Figure S12D. (E) Normalized G–V curves plotted against the membrane voltage for the WT and the mutants. They were taken from the tail currents: −20 mV for WT and +20 mV for the mutants. Data are presented as mean ± SEM. WT *n* = 5, W585A *n* = 5, N633A *n* = 4, where *n* is the number of biologically independent cells. For visualization purposes, the current traces were decimated to 20 μs.

Additionally, the I–V and G–V curves for W585A and N633A were strikingly different from those of the WT. Moreover, the G–V curves had two distinct voltage-dependent processes (Figure 4E and Tables 1 and 2). The second component in the G–V curve for W585A and N633A appeared in the voltage range of V > 0 mV where WT hERG inactivates/rectifies (Figure 4D–E). These results suggest that W585A and N633A not only prevent inactivation but also generate another conductive state. Moreover, they are different from the S631A case because this mutation removes inactivation by shifting its voltage dependence^44^ but does not generate a second voltage-dependent process in the G–V curve. The common trait, instead, is that in all cases, the channels exhibited an impaired transition toward the inactivated state. Overall, these experimental assays confirm the crucial role of W585 and N633 in hERG inactivation, in remarkable agreement with the computational analysis.

**Table 1:** Fitted Values for the G–V for WT hERG

	V_1/2_	z
WT	–14.9 ± 0.2	2.7 ± 0.0

**Table 2:** Fitted Values for the G–Vs for W585A and N633A

	G_1_	V_1_	z_1_	G_2_	V_2_	z_2_
**W585A**	0.4 ± 0.0	-32.4 ± 1.0	2.5 ± 0.2	0.7 ± 0.1	77.7 ± 3.7	0.8 ± 0.1
**N633A**	0.2 ± 0.0	-43.3 ± 0.9	2.9 ± 0.2	1.1 ± 0.0	93.8 ± 1.1	1.2 ± 0.0

## Discussion and Conclusions

In this work, C-type inactivation of the hERG K^+^ channel was investigated by employing a combination of equilibrium and nonequilibrium Molecular Dynamics (MD) simulations, together with site-directed mutagenesis and electrophysiological assays. Our equilibrium MD simulations demonstrate that the presence of K^+^ ions within the selectivity filter (SF), at least in key sites such as S3, stabilizes the open conformation and prevents its spontaneous inactivation. This observation is consistent with the experimental evidence that K_v_ channels exhibit reduced inactivation at high extracellular K^+^ concentrations^2,14,48–50^ and that K^+^ ions are required to exit the SF for inactivation to happen in hERG.^32^ More specifically, our data show that this transition can occur when the S3 binding site is unoccupied, even if the remaining sites are fully occupied (Figures S2–S4), consistent with previous studies.^11,14^ This suggests that S3 may contribute to controlling inactivation, although further computational and experimental work is necessary to confirm this hypothesis. Finally, simulations performed with AMBER and CHARMM36 force fields showed a somewhat different inactivation behavior, with CHARMM36 showing a greater tendency for inactivation. This evidence aligns with previous computational studies,^13,34,41^ highlighting the importance of the force field in studying ion channel dynamics.

Dynamical Nonequilibrium MD simulations^20,21,25^ (Figure 1C) allowed the characterization of the hERG C-type inactivation dynamics, suggesting the presence of three major steps (Figure 3). After the loss of K^+^ from the extracellular end of the filter,^32^ the first step involves the disruption of key interactions, such as F617–S631 and W585–N633; they show the greatest difference in contact fraction between the open and inactivated states (Figure 2B, Figure S9C). These early conformational changes create a cascade effect that destabilizes the filter architecture, thus determining channel inactivation by collapse. F617 and S631 are localized on the S5–P helix and on the extracellular side of the SF, respectively, and their interaction in the open state is mediated by a hydrophobic interaction between the side chain of F617 and the backbone of S631 (Figure S11). In previous work, we showed that F617A abolishes the channel expression, while F617L eliminates the inactivation, thus supporting the relevance of F617 in triggering this process.^46^ By decreasing the side chain of F617, e.g., with alanine or leucine mutations, the hydrophobic interaction is impaired, which in turn prevents the cascade of conformational changes that culminates with the inactivation. Similarly, S631 loses contact with F617 during inactivation and shows significant C_α_ displacement (Tables S1–S3). For example, S631A was found to attenuate inactivation;^47^ S631V, S631K, and S631E were found to abolish it; while S631Y exhibited no apparent effect.^51^ Perturbing the side chain of S631 by mutagenesis can shift its backbone either closer to or farther from the side chain of F617, thereby weakening or strengthening this interaction and consequently affecting the inactivated state of hERG. Indeed, when S631 was mutated to cysteine, reduction or oxidation of the thiol group produced distinct effects on inactivation, while modification with MTSET or MTSES disrupted both inactivation and ion selectivity.^52^ W585 and N633 are in contact via a hydrogen bond (Figure S11) which is lost during the transition from the open to the inactivated state. Based on this observation, one might expect that alanine mutations would enhance inactivation by favoring the inactivated state. However, experiments showed the opposite effect: in both W585A and N633A mutants, inactivation is disrupted. These effects can be explained by the disruption of a complex network of interactions ensuing from the introduction of the mutations. W585A ended up enhancing the interaction with N633 because substitution of tryptophan by alanine removes the bulky side chain that could be interacting with W568 (Figure S13), allowing the backbone at position 585 to be displaced toward N633. This prevents the disruption of the interaction between W585 and N633, which in turn impedes the onset of inactivation. W568 has been shown to be a part of the noncanonical chain of residues coupling the VSD to the PD, which plays a role in hERG inactivation.^46^ For N633A, a complementary explanation is that N633 does not merely serve as a passive hydrogen-bond acceptor but is an active structural element whose side chain participates in a broader interaction network that is both intra- and intersubunit at the SF–S5–P helix interface. Mutating N633 to alanine eliminates these contacts, uncoupling this interface and removing the mechanical basis for the conformational change rather than simply preempting it. In this view, the N633–W585 interaction is not just a latch that must be released but is part of the load-bearing architecture that drives and propagates the inactivation movement. Its absence, therefore, does not mimic the inactivated state but instead dismantles the pathway entirely. Nevertheless, the effect of the W585 and N633 mutations on hERG C-type inactivation reported in the present manuscript agrees with previous works showing that structural alterations of the S5–P helix and the extracellular loops of the pore domain affect such transition.^32,43,53^

The second and third steps of inactivation are constituted by flipping of V625 and constriction of SF, respectively. While these conformational changes have been previously reported,^12,14^ this work clarifies their causal link: V625 flipping occurs before SF constriction (Figure 3); the exit of K^+^ ions from the SF determines the initial rearrangement of the filter^32^ and thus represents the trigger for V625 flipping, and not the opposite. This finding aligns with the evidence that ion occupancy of the filter affects the gating of BK and K_ir_ channels^54–57^ and the C-type inactivation of KcsA, Shaker, hERG, and K2P channels.^31,58^

Previous works highlighted asymmetric protein rearrangements in the nonconducting SF of hERG^12^ during inactivation, like those of other K^+^ channels.^59^ Our equilibrium MD simulations show a strong asymmetry arising from the flipping of the V625 backbone carbonyls, occurring mostly in three out of four subunits (Figure S3). Consistently, when the filter collapses, the G626-G626 C_α_ distance of two opposite subunits is normally ∼2 Å greater than that of the other subunits (Figure S4).^12^ Given the relationship between V625 flipping and filter constriction, the number of V625 residues adopting the flipped conformation in each channel during the multiple short D-NEMD simulations indicates that these nonequilibrium simulations can also capture asymmetry associated with hERG inactivation (Figure S14). However, averaged quantities, such as the V625 angle or the G626 distance reported in Figures S7 and S9A, may obscure this heterogeneity because they combine measurements from different subunits.

The voltage represents the driving force for C-type inactivation due to its effect on the ions in the filter. In our D-NEMD simulations at +900 mV, the inactivation occurred after 75 ns (Figure S9), thus being accelerated compared to the millisecond time scale at physiological voltages. This extreme nonphysiological voltage was used to accelerate the sampling of conformational transitions within computationally accessible time scales,^60^ facilitating ion depletion from the SF. This stimulus results in the destabilization of the carbonyl oxygen-ion interactions in the filter that maintain the open state, thereby promoting the inactivation process. Conversely, at lower voltages (+500 mV and +700 mV), no ion movements were seen during 500-ns simulations, accounting for much slower inactivation, in line with experiments. However, conduction remains strongly influenced by the initial SF configuration even in the presence of an applied voltage.^60^ Therefore, the behavior observed in our simulations may depend on the starting configuration, namely, the stable configuration obtained at 0 mV (WWWKK). Despite this potential dependence, the relationship between the applied voltage and the inactivation rate suggests that our findings capture the fundamental mechanistic steps of the process, albeit on an accelerated time scale.

Overall, the present findings suggest a possible pathway that leads to C-type inactivation of the hERG channel, providing an atomistic explanation for the mutation effects of key residues involved in the process. From a methodological point of view, the application of D-NEMD simulations demonstrates the power of this approach to investigate rapid, nonequilibrium biological processes that are difficult to capture via experiments and standard molecular dynamics approaches.

## Methods

### MD Simulations

The hERG open state used in Bassetto et al., 2023 ^46^ was generated from the experimentally solved structure (PDB: 5VA2)^26^ with K^+^ ions in the S1 and S3 ion sites. Using the CHARMM-GUI membrane builder and its equilibration protocol,^61,62^ it was embedded in a bilayer of 1010 1-palmitoyl-2-oleoyl-*sn*-glycero-3-phosphocholine (POPC) lipids and solvated with 49,467 TIP3P water molecules^63^ and 0.15 M KCl to form a simulation box of ca. 125 × 125 × 150 Å, totaling 223,584 atoms. The WHAT-IF server^64^ was used to analyze the protonation states of residues. All aspartates and glutamates were ionized. H402, H739, H771, H831, and H834 were predicted to be in the δ protonation state; H485, H492, H562, H578, H587, H674, H687, H703, H731, H762, and H851 were assigned to the ϵ protonation state. The resulting equilibrated system—where the equilibration was assessed via RMSD calculation (Figure S15)—with K^+^ ions in S3 and S4 was used as the initial configuration for equilibrium simulations with different conditions: the voltage (V_z_) along the whole length of the *z*-axis of the simulation box was set to 0 mV, +500 mV, +700 mV, and +900 mV to simulate the depolarized membrane potential; the K^+^ ions in S3 and S4 were maintained in the so-called “2 K^+^” systems, while they were manually removed in the “0 K^+^” systems; in the “AMBER” simulations, the ff14SB force field was used for the protein^65^ and Lipid17 for the lipids^66^ while for the “CHARMM36” simulations, the CHARMM36 force field was employed.^67,68^ Multiple independent replicas were run (Table S5), thus amounting to a total simulation time of 6.95 μs. D-NEMD simulations were run^21^ starting from the 2 K^+^ open state equilibrated at 0 mV. Coordinates and velocities were extracted every 2 ns from the last 40 ns of the equilibrium trajectories, providing a total of 20 configurations for D-NEMD initiation, which were run for 25 ns each, thus totaling 500 ns. The K^+^ ions from the equilibrated SF were removed as a perturbation. As a control, D-NEMD simulations were also run starting from the same structure, but applying a voltage of +900 mV as a perturbation. In this case, only 4 configurations were simulated for 150 ns each, thus totaling 600 ns. All D-NEMD simulations were run only with the CHARMM36 force field^67,68^ due to its intrinsic capability to induce structural modification of the filter in a simulation time less than AMBER.^41^ C_α_ deviation values were averaged over all 20 nonequilibrium simulations per system, and the standard deviations and standard error of the mean were calculated using the Kubo–Onsager relation.^20,22,25,69^ Both equilibrium and D-NEMD simulations were run with NAMD 3.0.^70^ The pressure was kept at 1.01325 bar by the Nosé–Hoover Langevin piston method^71^ and the temperature was maintained at 303.15 K by a Langevin thermostat with a damping coefficient of 1 ps^–1^. Long-range electrostatic interactions were evaluated with the smooth Particle Mesh Ewald algorithm^72^ with a grid space of 1 Å. For short-range nonbonded interactions, a cutoff of 12 Å with a switching function at 10.0 Å was used. The integration time step was 2 fs.

### Computational Analysis

The representative structures (*n = 5*) of the SF conformations (from S624 to G628) sampled during the equilibrium simulations where the channels reached an inactivated configuration of the SF, i.e., V625 flipped in at least three subunits and the SF constricted, were identified using the CPPTRAJ program of the AMBER package with the K-means clustering algorithm.^73,74^ Precisely, according to Figures S3–S4, the chunks of the simulations considered were: AMBER 0 K^+^ 0 mV replica 3 from 550 ns; CHARMM36 0 K^+^ 0 mV replica 1 from 220 ns, replica 2 from 250 ns; AMBER 2 K^+^ +900 mV replica 1 from 40 ns, replica 3 from 200 ns; CHARMM36 2 K^+^ +900 mV replica 1 from 200 ns, replica 2 from 80 ns, replica 3 from 50 to 150 ns, replica 4 from 150 ns. An RMSD-based metric considering all the SF’s residues was used to evaluate the pairwise distance between conformations. The pore radius profile was measured using the HOLE program.^75^ The silhouette score^76^ was used to measure cluster quality (Figure S16). The angle of the SF backbone carbonyls was calculated as the angle between the projection on the xy-plane of the vector between the backbone carbonyl C and the backbone carbonyl O, and the projection on the xy-plane of the vector between the backbone carbonyl C and the center of mass of the SF.^14^ The constriction of the SF was quantified by monitoring the distance of C_α_ of G626 of opposite subunits, where values less than 5 Å defined a constricted filter.^12^ The bending angle of helix S6 (θ) was studied as in our previous work:^77^

1 $$\theta =arc\cos\left(\frac{{\mathrm{v⃗}}_{\mathrm{CT}}\cdot {\mathrm{v⃗}}_{\mathrm{CB}}}{\left|{\mathrm{v⃗}}_{\mathrm{CT}}\right|\left|{\mathrm{v⃗}}_{\mathrm{CB}}\right|}\right)$$

where v⃗_CT_ is a vector pointing from the middle region of helix S6 (residues 646–650) to the top of the same helix (residues 635–639). Similarly, v⃗_CB_ is a vector pointing from the middle region to the bottom end of helix S6 (residues 663–667). As reference points, the bending angle of helix S6 in the open state (PDB: 5VA2 ^26^) and in the inactivated state (PDB: 9CHQ^14^) were computed, θ = 152.55^◦^ and θ = 152.35^◦^, respectively. Since no structure of hERG in the closed state is available, we considered the closed state of the homologous EAG1 channel (PDB: 8EP1 ^35^), for which θ = 165.74^◦^, where v⃗_CT_ is a vector pointing from the middle region of helix S6 (residues 458–462) to the top of the same helix (residues 447–451) and v⃗_CB_ is a vector pointing from the middle region to the bottom end of helix S6 (residues 474–478). Intrasubunit interactions were identified via semibinary contact maps (C_ij_), which were computed with a truncated Gaussian kernel:

2 $$K\left(d_{\mathrm{ij}}\right)=\{\begin{array}{l} 1,d_{\mathrm{ij}}\leq c\\ e^{-\left(d_{\mathrm{ij}}^{2}-c^{2}\right)/2\sigma^{2}},d_{\mathrm{ij}}>c \end{array}$$

where d_ij_ is the distance between heavy atoms of the i and j amino acids and c is the cutoff distance set to 4.5 Å. The width σ of the Gaussian kernel was chosen to attain a negligibly small value of the kernel at d_cut_ = 8 Å. So, we imposed K­(d_cut_) = 10^‑5^ attaining σ = 0.138.^78,79^ The contact map was computed by averaging the value of the kernel over all the frames of the trajectory:

3 $$C_{\mathrm{ij}}=\frac{1}{N_{\mathrm{frames}}}\overset{N_{\mathrm{frames}}}{\underset{n=1}{\sum }}K\left(d_{\mathrm{ij}}\left(n\right)\right)$$

A contact fraction (CF) equal to 1 corresponds to the presence of the interaction, while a contact fraction equal to 0 corresponds to the absence of that interaction. Directional displacements were explicitly retained and quantified through Cartesian displacement components (Δx, Δy, Δz) for each C_α_ atom. Each trajectory replica was first structurally aligned to its reference frame to remove global roto-translational motions. After alignment, the Cartesian coordinates of each C_α_ atom at a given time point (t) were compared with those of the aligned initial frame (t = 0). The displacement vector was computed as Δr­(t) = r­(t) – r(0), where r­(t) = (x­(t), y­(t), z­(t)) and r(0) = (x_0_, y_0_, z_0_) correspond to the Cartesian coordinates of the same C_α_ atom at time *t* and in the aligned reference structure, respectively. The signed Cartesian displacement components were then calculated as Δx = x­(t) – x_0_, Δy = y­(t) – y_0_ and Δz = z­(t) – z_0_, thereby preserving directional information along the simulation axes. The simulations were visually inspected using VMD 2.0.^80^

### Site-Directed Mutagenesis

We used the human hERG K^+^ channel cloned into the SP64 vector. Mutations were performed using Quik-Change, together with custom primers from Integrated DNA Technologies (Integrated DNA Technologies, Inc., Coralville, IA). hERG cDNA and its mutants were sequenced to ensure accurate DNA sequencing, linearized by endonuclease *Eco*RI (New England Biolabs, Ipswich, MA), and cleaned up with a NucleoSpin Gel and PCR Clean-up Kit (Macherey–Nagel, Bethlehem, PA). *In vitro* transcription kits were used to transcribe cDNA and generate cRNA (SP6 RNA expression kit; Ambion Invitrogen, Thermo Fisher Scientific, Waltham, MA).

### Oocytes Preparation

*Xenopus laevis* oocytes were purchased from XENOPUS1 (Dexter, MI). The follicular membrane was digested using collagenase type 2 (Worthington Biochemical Corporation, Lakewood, NJ) at 2 mg/mL and supplemented by bovine serum albumin (BSA). Following the follicular membrane digestion, oocytes were incubated in standard oocytes solution (SOS) containing, in mM: 96 NaCl, 2 KCl, 1.8 CaCl_2_, 1 MgCl_2_ (Sigma-Aldrich, St. Louis, MO), 0.1 EDTA (Promega, Madison, WI), 10 HEPES, and pH set to 7.4 with NaOH. SOS was supplemented with 50 μg/mL gentamycin. After 12 h of digestion, defolliculated oocytes stage V–VI were injected with cRNA (50 ng diluted in 50 nL of RNase-free water) and incubated at 18 °C for 3 days prior to recording. Unless otherwise stated, chemicals were purchased from Fisher Scientific (Fair Lawn, NJ).

### Electrophysiological Recordings

Ionic currents were recorded using the cut-open oocyte voltage-clamp (COVC) method.^81^ Currents were acquired by a setup comprising a Dagan CA-1B amplifier (Dagan, Minneapolis, MN) with a built-in low-pass 4-pole Bessel filter. A 16-bit A/D converter (USB-1604, Measurement Computing, Norton, MA) was used by acquisition and controlled by in-house software (GPatch64MC). Data were sampled at 1 MHz, digitally filtered at the Nyquist frequency and decimated for the desired acquisition rate. Capacitive transient currents were compensated using a dedicated circuit. The voltage-measuring pipet was pulled using a horizontal puller (P-1000 Model, Sutter Instruments, Novato, CA). For ionic current measurements, the external solution was composed of (mM): KOH 12, CaOH_2_, HEPES 10, EDTA 0.1, NMDG (*N*-Methyl-d-glucamine) 108, pH 7.4 (adjusted with MES-Methanesulfonic acid). The internal solution was composed of (mM): KOH 120, EGTA 2, HEPES 10, pH 7.4 (adjusted with MES- Methanesulfonic acid). The holding potential was set to −80 mV. Recordings were performed at room temperature (17, 18 °C).

### Data Analysis

The voltage dependence of the channel conductance was taken by tail currents. After long depolarizing pulses to different membrane potentials (P1), the membrane was set to a hyperpolarized voltage of −20 mV for WT and 20 mV for the mutants (P2). The currents elicited by P2 were normalized to the maximum of each experiment, averaged, and plotted against the membrane voltage of pulse P1 to obtain a conductance–voltage (G–V) curve. The G–V curves for the WT were fitted using a two-state model with the following equation:

4 $$G\left(V_{m}\right)=\frac{1}{1+\exp\left(\frac{zF}{RT}\left(V_{1/2}-V_{m}\right)\right)}$$

where z is the apparent valence of the charge times the fraction of the electric field of the transition and V_1/2_ is the voltage at 50% of the maximal conductance. Since the mutants had two components in their G–V curves, we used the sum of two Boltzmann distributions^82^ in the form:

5 $$G\left(V_{m}\right)=\frac{G_{1}}{1+\exp\left(\frac{z_{1}F}{RT}\left(V_{1}-V_{m}\right)\right)}+\frac{G_{2}}{1+\exp\left(\frac{z_{2}F}{RT}\left(V_{2}-V_{m}\right)\right)}$$

where G_1_ and G_2_ are the fractional conductance for the first and second components of the G–V, V_1_ and V_2_ are the voltage for 50% of the fractional conductance, respectively, and z_1_ and z_2_ are the apparent valence of the charge times the fraction of the electric field of the first and second transitions, respectively. Origin 2024b (OriginLab Corporation, Northampton, MA) was used for calculating G–Vs, plotting, and fitting the data. Data analysis was also performed using a program written in-house (Analysis).

## Supplementary Material











## Data Availability

The topology files, the starting atomic coordinates, and an example configuration file are openly available in ZENODO at 10.5281/zenodo.18809243.

## Notes

Side view of the structural representation of the Cartesian coordinates of C_α_ displacement extracted from the ensemble D-NEMD simulations after 25 ns from the removal of K^+^ ions in the SF (MP4)
Top view of the structural representation of the Cartesian coordinates of Cα displacement extracted from the ensemble D-NEMD simulations after 25 ns from the removal of K+ ions in the SF (MP4)
Formation and breaking of contacts between residues on the SF and P-helix in the ensemble of 25 ns D-NEMD simulations where the removal of the ions was used as a perturbation (MP4)
Formation and breaking of contacts between residues on the SF and P-helix in the ensemble of 150 ns D-NEMD simulations where the high voltage at 900 mV was used as a perturbation (MP4)
Supporting Figure 1–16: conformational transitions occurring during equilibrium MD simulations of the hERG WT open state at 0 mV; axial positions of ions in the SF in equilibrium MD simulations of the hERG WT open state under different initial conditions and simulation parameters; V625 conformational transitions in equilibrium MD simulations under varied conditions; G626 distance profiles in equilibrium MD simulations under varied conditions; bending angle of S6 helices delimiting the hERG pore; cluster analysis performed on the residues of the SF in the equilibrium MD simulations; profiles of V625 rotation and distance between G626 of two opposite subunits computed in the ensemble of 25 ns D-NEMD simulations with the removal of K^+^ as perturbation; axial positions of ions in the SF in equilibrium MD simulations of the hERG WT open state at +500 mV and +700 mV; V625 rotation, distance between G626 of two opposite subunits, and contact maps showing intrasubunit interactions in D-NEMD simulations with the voltage of +900 mV applied as a perturbation; hydrogen bonds between Y616–N629, S620–V625, and S620–V625 in the ensemble D-NEMD simulations after the K^+^ removal and the application of +900 mV as perturbations; representative snapshot showing F617–S631 and W585–N633 interactions in the open state at the beginning of the D-NEMD simulations; long depolarizing pulses show that W585A and N633A do not inactivate; role of W568 in hERG inactivation; number of V625 flipped at the end of the D-NEMD simulations; root-mean-square displacement profile of the hERG system run at 0 mV with the WKWKW SF configuration; and silhouette scores of the cluster analysis. Supporting Tables 1–5: C_α_ displacement of the protein amino acids after 5, 10, 15, 20, and 25 ns of the K^+^ ions removal in the D-NEMD simulations; Cartesian coordinates of C_α_ displacement extracted from the ensemble D-NEMD simulations after 25 ns from the removal of K^+^ ions in the SF; C_α_ displacement of the protein amino acids after 30, 60, 90, 120, and 150 ns of the electric field in the D-NEMD simulations; Cartesian coordinates of C_α_ displacement extracted from the ensemble D-NEMD simulations after 150 ns from the application of the +900 mV electric field; and summary of the equilibrium MD simulations considered in this study with the relevant features (PDF)
