# Supplementary material for: Dynamical Nonequilibrium Molecular Dynamics Simulations Reveal Atomistic Steps of C‑Type Inactivation in Cardiac hERG Channels
Source: J Am Chem Soc. 2026 Aug 25;148(35):37738–50. doi: 10.1021/jacs.6c05939 (PMC13564440; doi:10.1021/jacs.6c05939)
Supplement: Supplementary file 1 [file ja6c05939_si_005.pdf]

SUPPORTING INFORMATION FOR

Dynamical Nonequilibrium Molecular Dynamics Simulations Reveal Atomistic Steps of C-type Inactivation in Cardiac hERG Channels

Flavio Costa [1], Carlos Bassetto [2]*, Francisco Bezanilla [3,4,5] and Alberto Giacomello [1]*

[1] Dipartimento di Ingegneria Meccanica e Aerospaziale, Sapienza Università di Roma, Rome, 00184, Italy.

[2] Department of Physics and Astronomy, UT San Antonio, San Antonio, TX, 78249, USA.

[3] Department of Biochemistry and Molecular Biology, The University of Chicago, Chicago, IL, 60637, USA.

[4] Institute for Biophysical Dynamics, The University of Chicago, Chicago, IL, 60637, USA.

[5] Centro Interdisciplinario de Neurociencias, Facultad de Ciencias, Universidad de Valparaiso, Valparaiso, 2284440, Chile.

* Corresponding author(s).

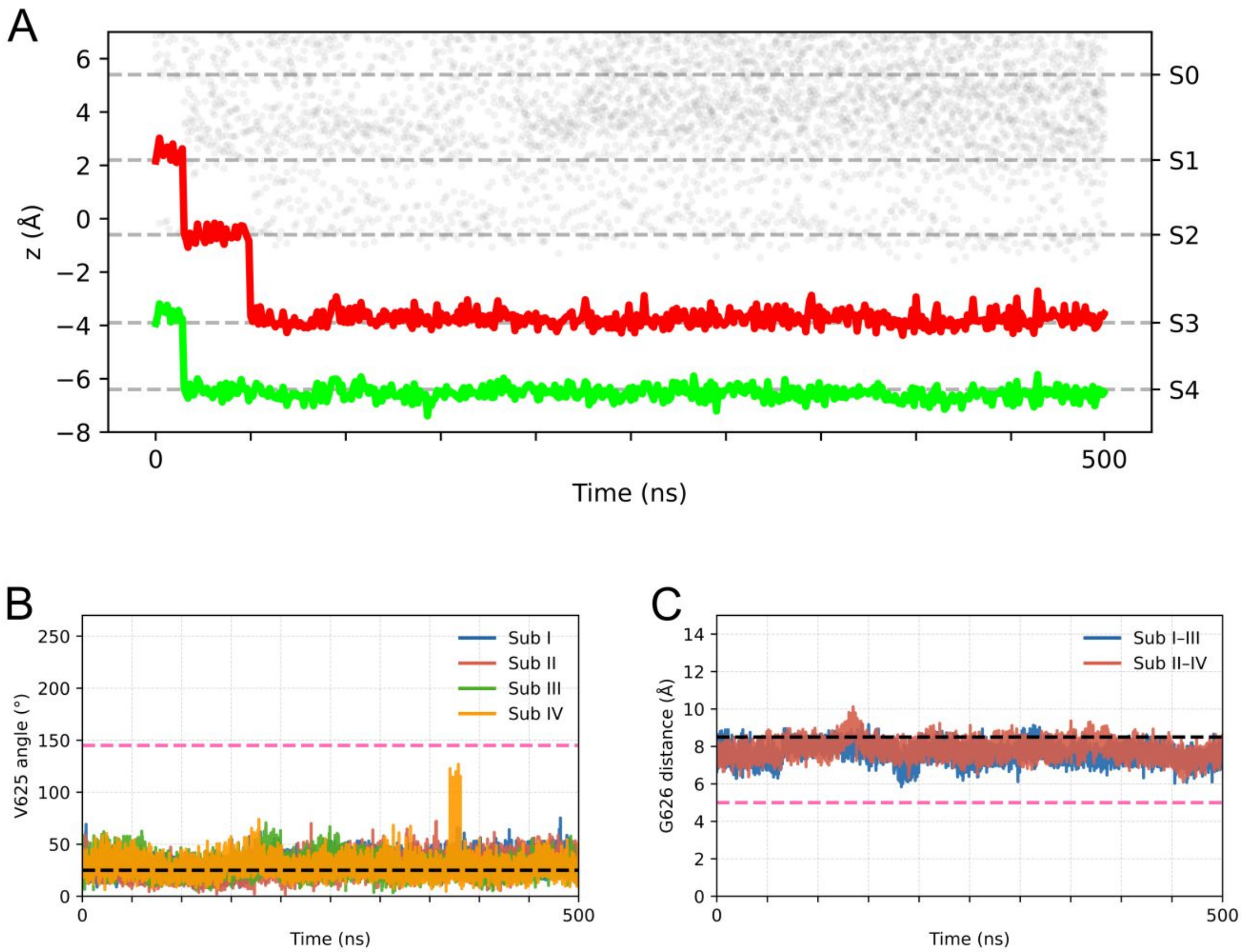

Figure S1. Conformational transitions occurring during equilibrium MD simulations of the hERG WT open state at 0 mV. (A) Axial positions of ions in the SF; different colors represent different potassium ions, and horizontal dashed lines indicate the binding sites S0 to S4. Water molecules are indicated by gray points. (B) Rotation angle of V625 and (C) distance measurements for G626 of two opposite subunits. Horizontal dashed lines show mean V625 rotation angles and G626 distance for the open state (black) and inactivated state (pink). Ticks on x-axis are spaced every 50 ns.

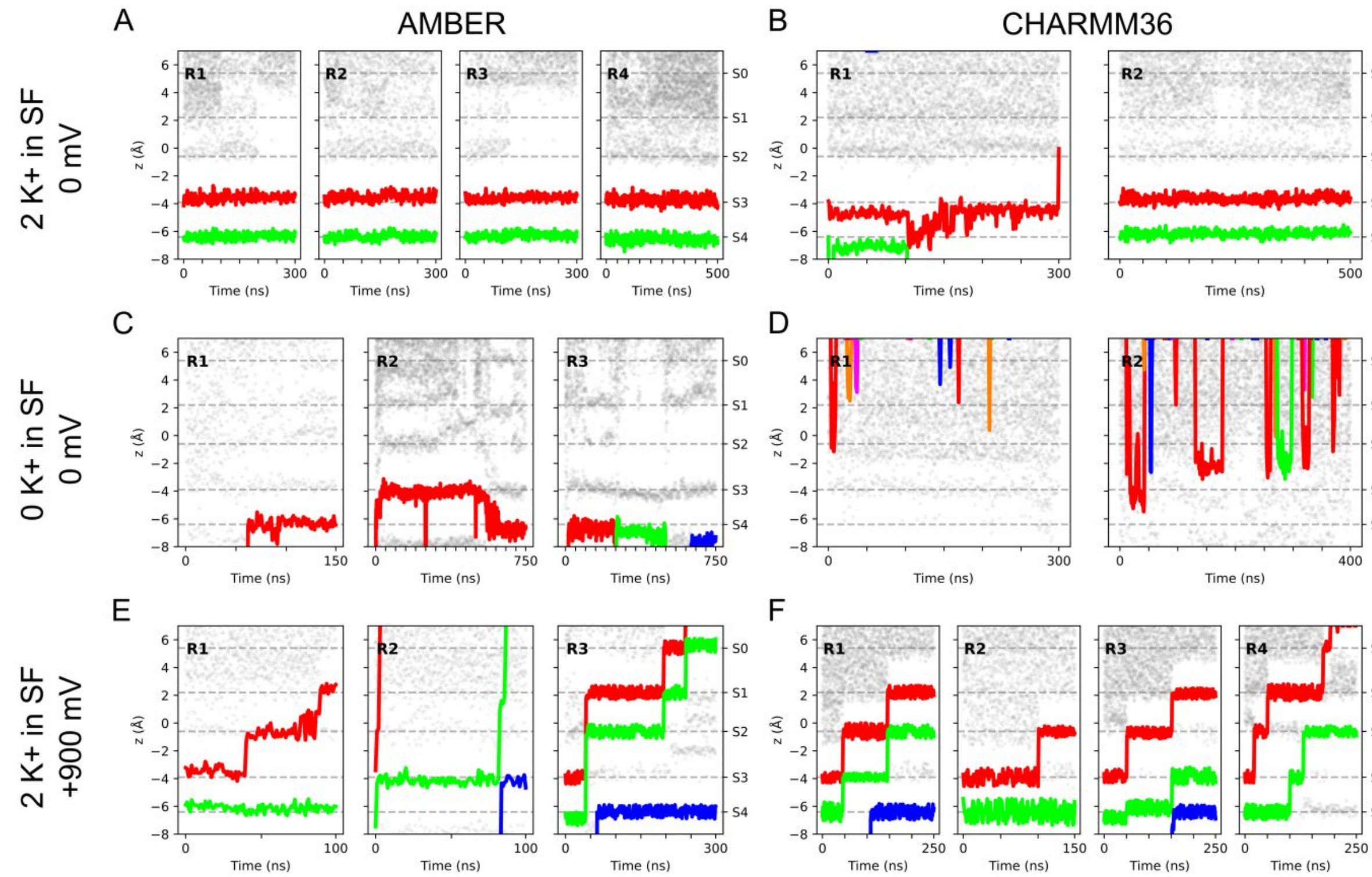

Figure S2. Axial positions of ions in the SF in equilibrium MD simulations of the hERG WT open state under different initial conditions and simulation parameters: (A-B) AMBER and CHARMM36 force fields with 2 K^+ ions at 0 mV, (C-D) AMBER and CHARMM36 force fields with 0 K^+ ions at 0 mV, (E-F) AMBER and CHARMM36 force fields with 2 K^+ ions at +900 mV. Different colors represent different potassium ions while water molecules are shown as grey points. "R" indicates the number of independent replicas; horizontal dashed lines represent the binding sites S0 to S4. Ticks on x-axis are spaced every 50 ns.

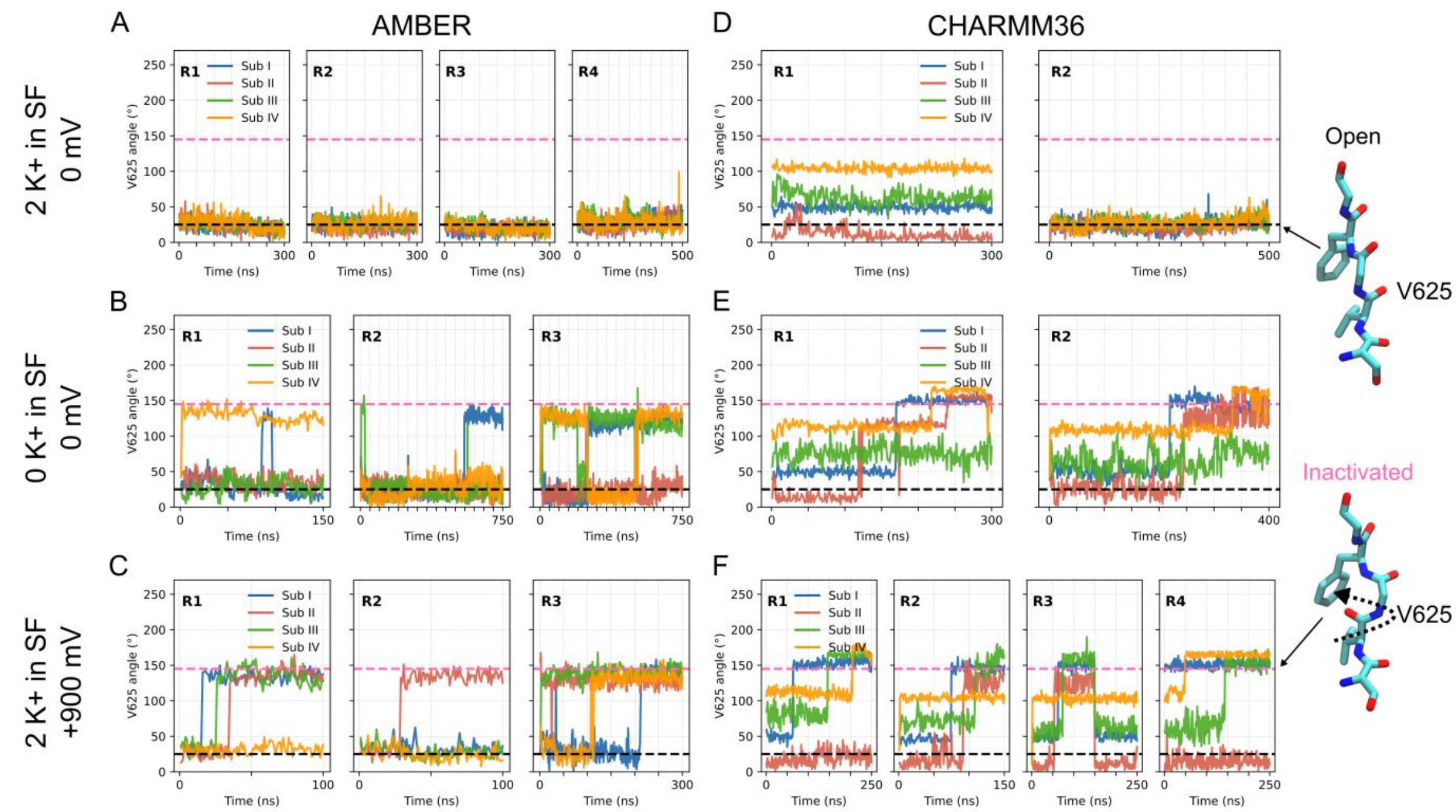

Figure S3. V625 conformational transitions occur spontaneously in equilibrium MD simulations under varied conditions. Rotation angle of V625 over simulation time for the open state under different initial conditions and simulation parameters: (A–C) AMBER force field with 2 K^+ ions (A), 0 K^+ ions (B), or 2 K^+ ions at +900 mV (C); (D–F) CHARMM36 force field with 2 K^+ ions (D), 0 K^+ ions (E), or 2 K^+ ions at +900 mV (F). All simulations performed at 0 mV unless otherwise noted. "R" indicates the number of independent replicas; ticks on x-axis are spaced every 50 ns. Horizontal dashed lines show mean V625 rotation angles for the open state (black) and inactivated state (pink), with representative structures of a subunit shown.

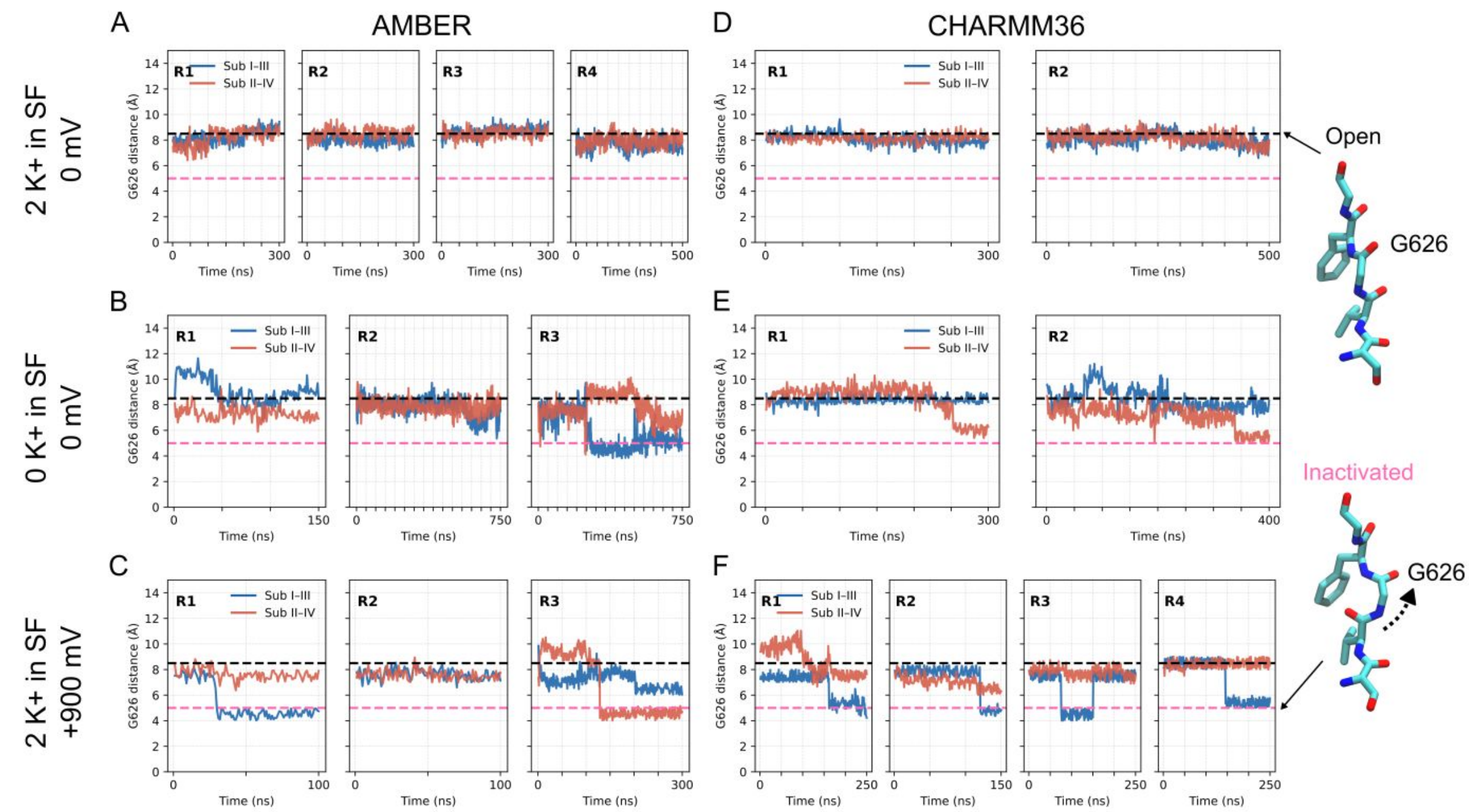

Figure S4. G626 distance profiles reveal conformational changes across simulation conditions. Distance measurements for G626 over simulation time for the open state under different initial conditions and simulation parameters: (A–C) AMBER force field with 2 K^+ ions (A), 0 K^+ ions (B), or 2 K^+ ions at +900 mV (C); (D–F) CHARMM36 force field with 2 K^+ ions (D), 0 K^+ ions (E), or 2 K^+ ions at +900 mV (F). All simulations performed at 0 mV unless otherwise noted. "R" indicates the number of independent replicas; ticks on x-axis are spaced every 50 ns. Horizontal dashed lines show mean G626 distances for the open state (black) and inactivated state (pink), with representative structures of a subunit shown.

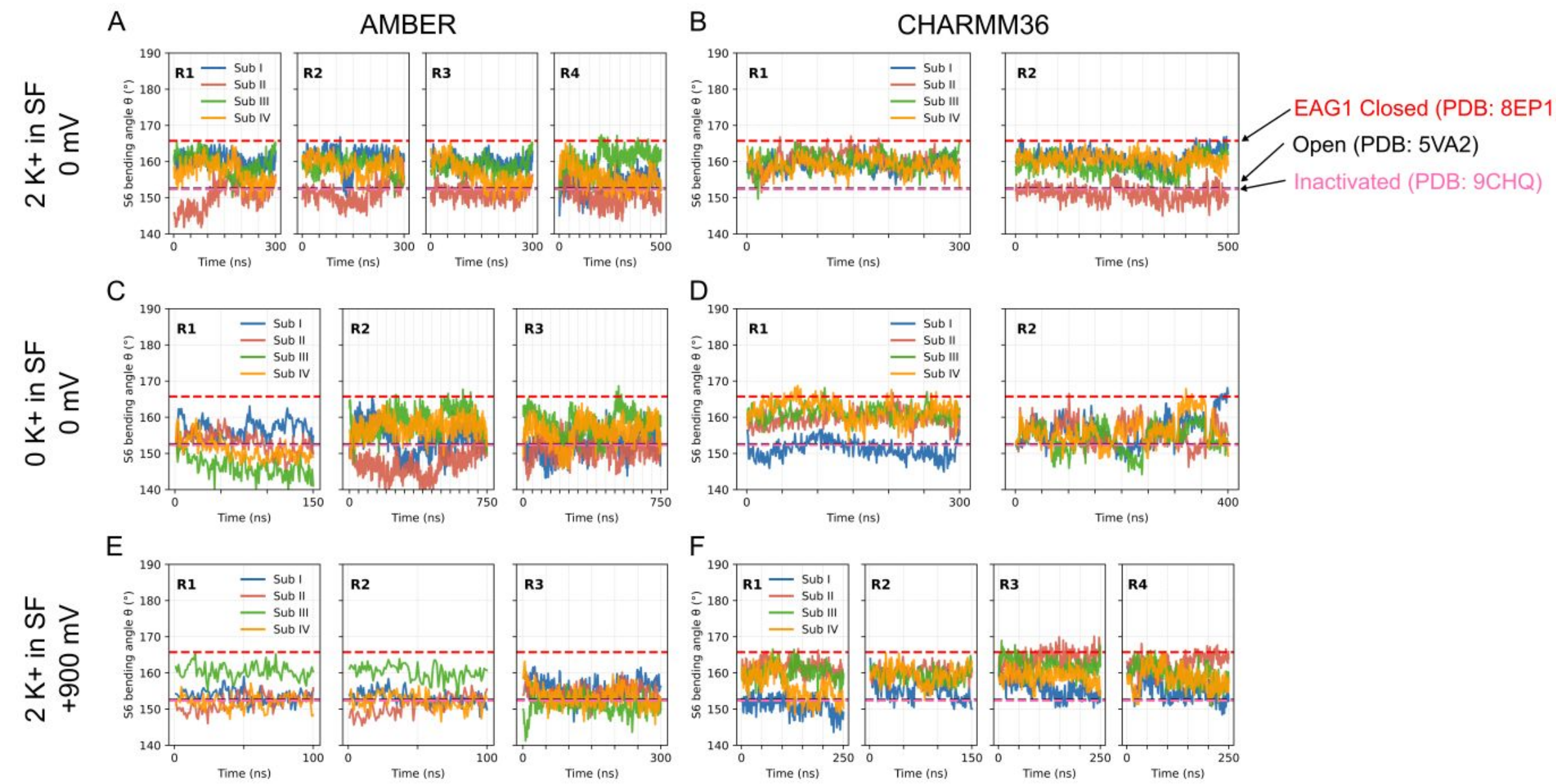

Figure S5: Bending angle of S6 helices delimitating the hERG pore. (A-B) AMBER and CHARMM36 force fields with 2 K^+ ions at 0 mV, (C-D) AMBER and CHARMM36 force fields with 0 K^+ ions at 0 mV, (E-F) AMBER and CHARMM36 force fields with 2 K^+ ions at +900 mV. The dashed black line refers to the value of the helix S6 in the open structure (PDB: 5VA2); the dashed pink line to that in the inactivated structure (PDB: 9CHQ); the dashed red line to that of the closed pore of the homologous EAG1 channel (PDB: 8EP1). Ticks on x-axis are spaced every 50 ns.

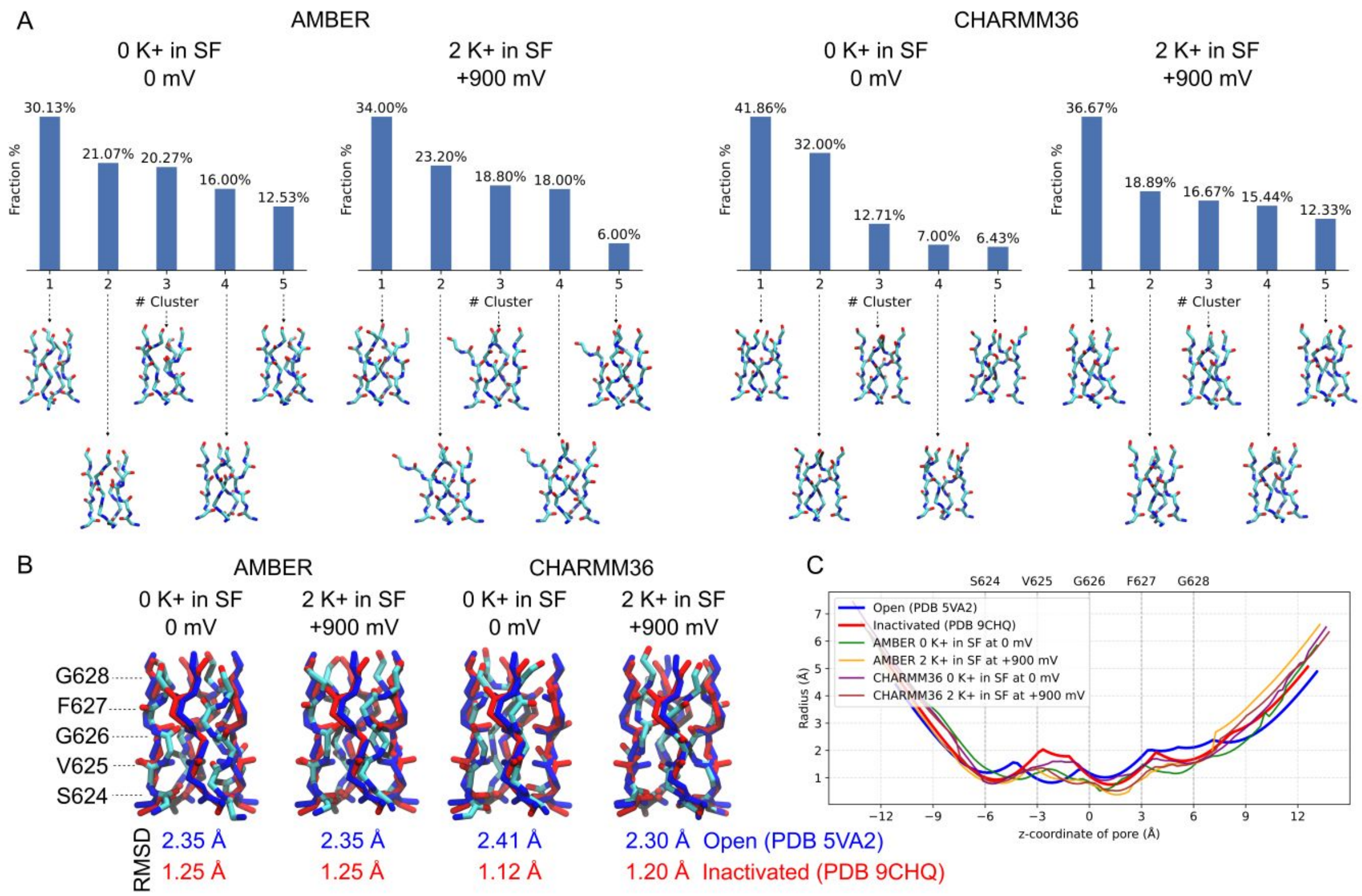

Figure S6. (A) Cluster analysis performed on the residues of the Selectivity Filter (SF) in the equilibrium MD simulations. For each cluster (n=5), snapshots of the most representative SF conformation are reported. (B) Comparison between the SF of the low-K+ hERG inactivated structure (PDB 9CHQ, in red), the open state (PDB 5VA2, in blue), and our computationally equilibrated inactivated systems of the most populated clusters (i.e., clusters #1). The RMSD values reported below the structures are computed considering all non-hydrogen atoms of the SF residues S624-G628. (C) Radius profile of the SF of the systems under consideration.

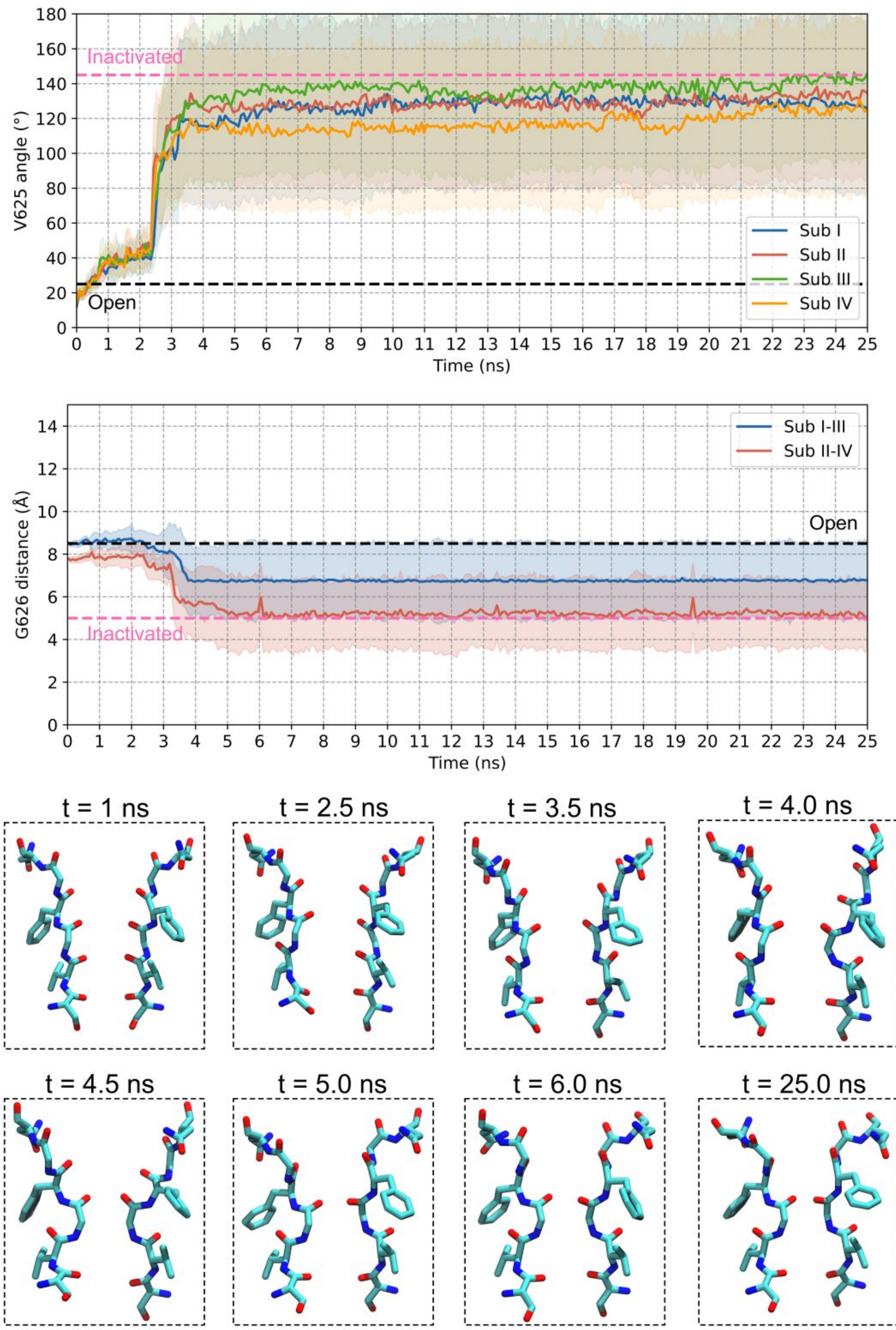

Figure S7. Profiles of V625 rotation and distance between G626 of two opposite subunits computed in the ensemble 25 ns D-NEMD simulations. The values correspond to the mean over the replicas while shaded region to the standard deviation. Horizontal dashed lines show mean V625 rotation angles and G626 distance for the open state (black) and inactivated state (pink).

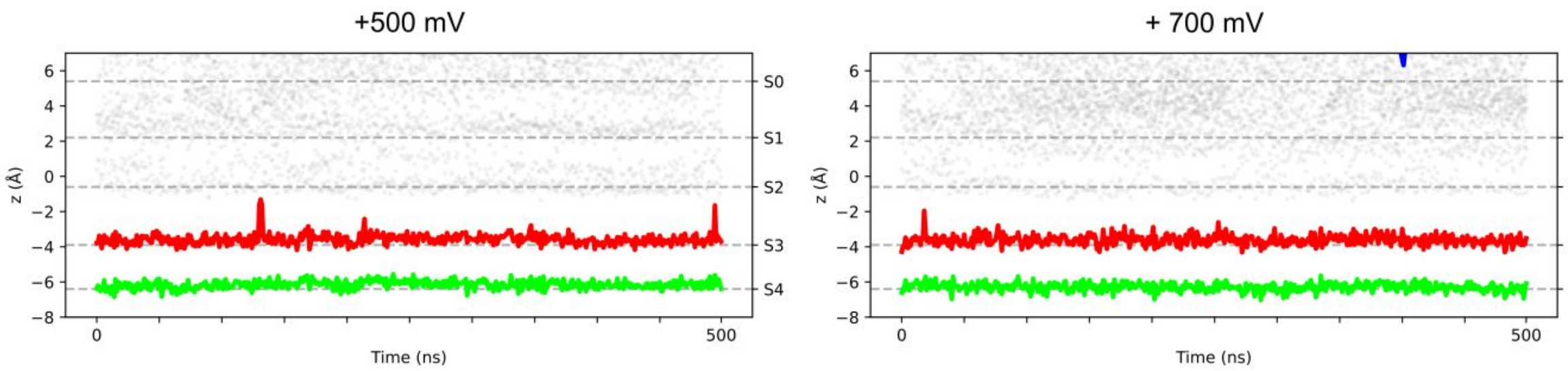

Figure S8. Axial positions of ions in the SF in equilibrium MD simulations of the hERG WT open state at +500 mV and +700 mV. Different colors represent different potassium ions while water molecules are shown as grey points. Horizontal dashed lines indicate the binding sites S0 to S4. Ticks on x-axis are spaced every 50 ns.

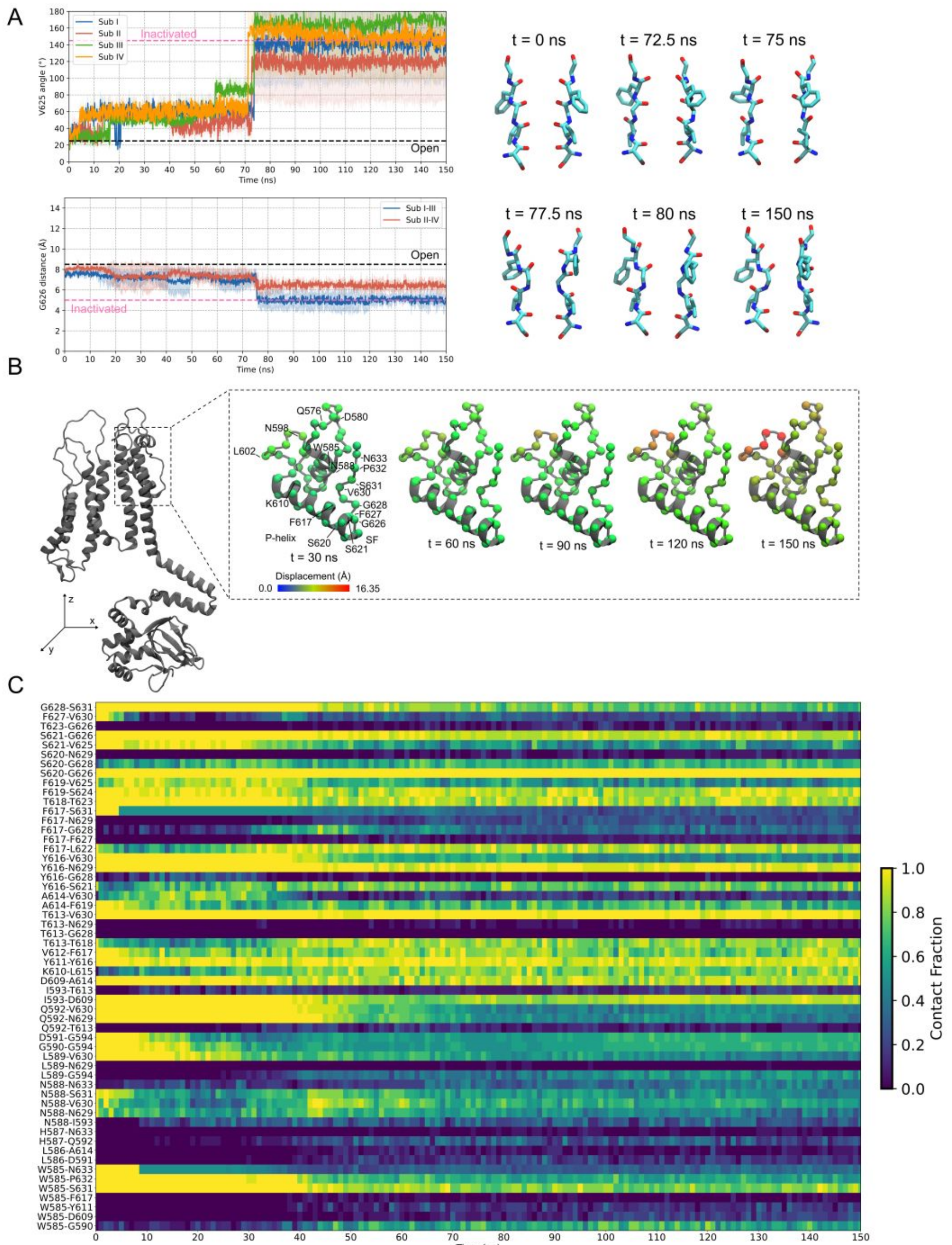

Figure S9. (A) Profiles of V625 rotation and distance between G626 of two opposite subunits computed in the ensemble 150 ns D-NEMD simulations applying the voltage +900 mV as perturbation. The values correspond to the mean over the four subunits and all the replicas, while shaded region to the standard deviation. Horizontal dashed lines show mean V625 rotation angles and G626 distance for the open state (black) and inactivated state (pink). (B) Average C_α displacement of the protein amino acids computed for each residue every 30 ns after the perturbation, over the four protein subunits, in the ensemble 150 ns D-NEMD simulations. Only the C_α of the residues are shown. (C) Contact map showing intra-subunit interactions computed in

the ensemble D-NEMD simulations where a contact fraction equal to 1 corresponds to the presence of the interaction between pairs of residues, while equal to 0 in its absence.

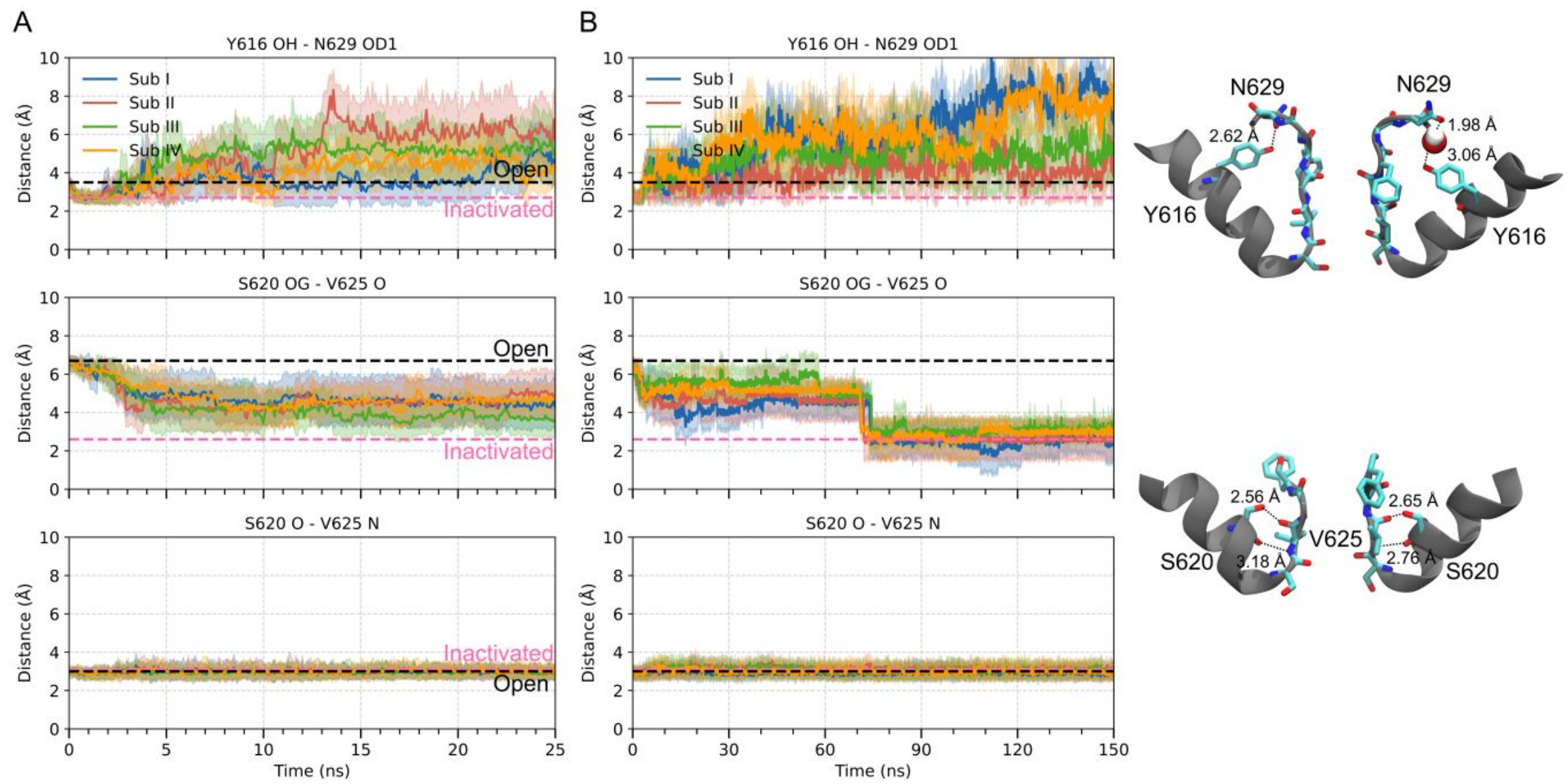

Figure S10: Hydrogen bonds between Y616-N629, S620-V625 and S620-V625 in the ensemble D-NEMD simulations after the K+ removal (A) or the application of +900 mV voltage (B). Horizontal dashed lines show the distance found in the open state (PDB 5VA2) and inactivated state (PDB 9CHQ). Representative snapshots at the end of 25 ns K^+-removal D-NEMD simulations where the protein residues are shown in licorice and the water molecule in VdW.

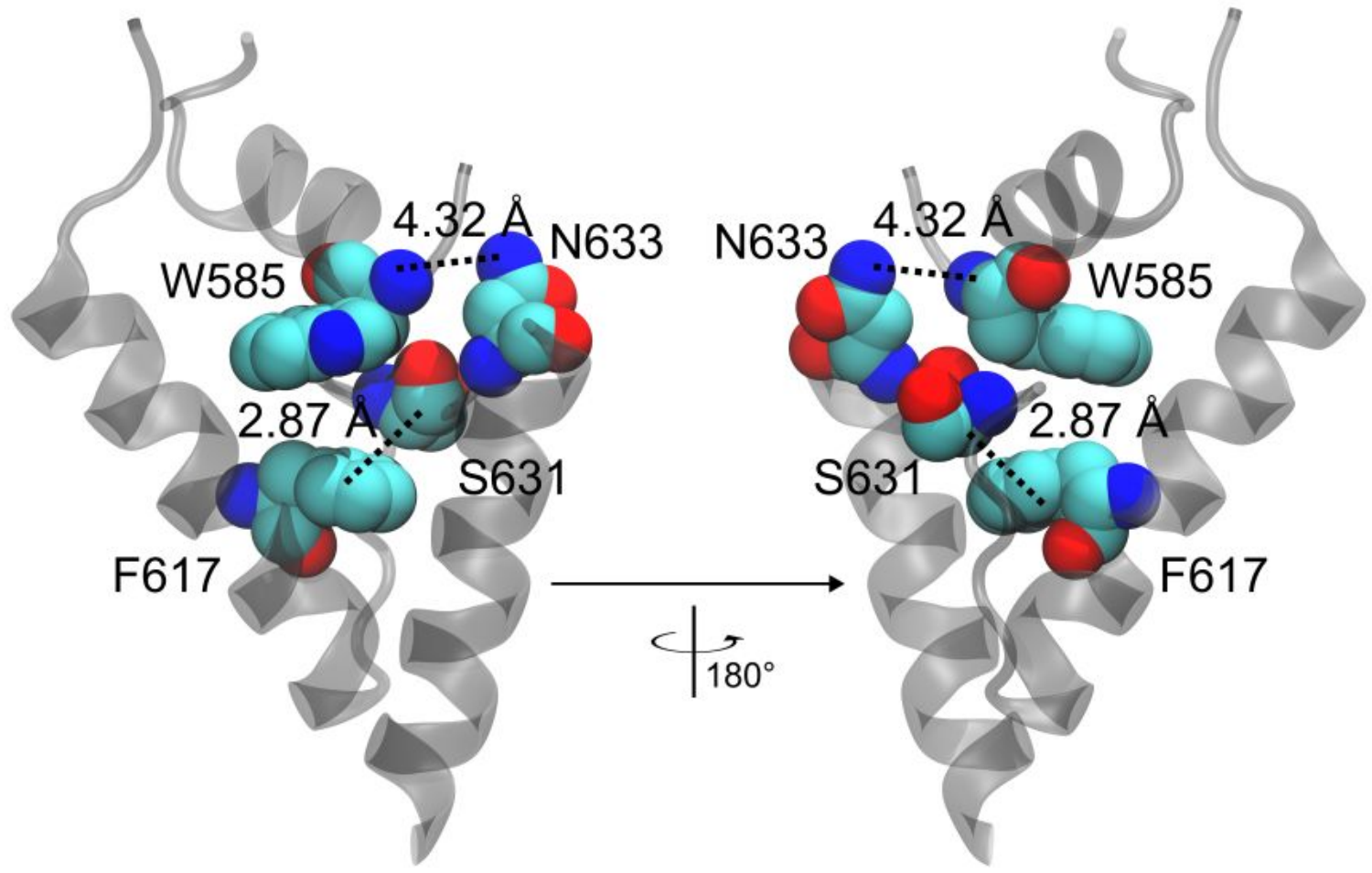

Figure S11: Representative snapshot showing F617-S631 and W585-N633 interactions in the open state. F617-S631 is mediated by a packing hydrophobic interaction between the bulky aromatic side chain of F617 and the backbone of S631; W585-N633 is a hydrogen bond between the nitrogen of the W585 backbone and N633 side chain.

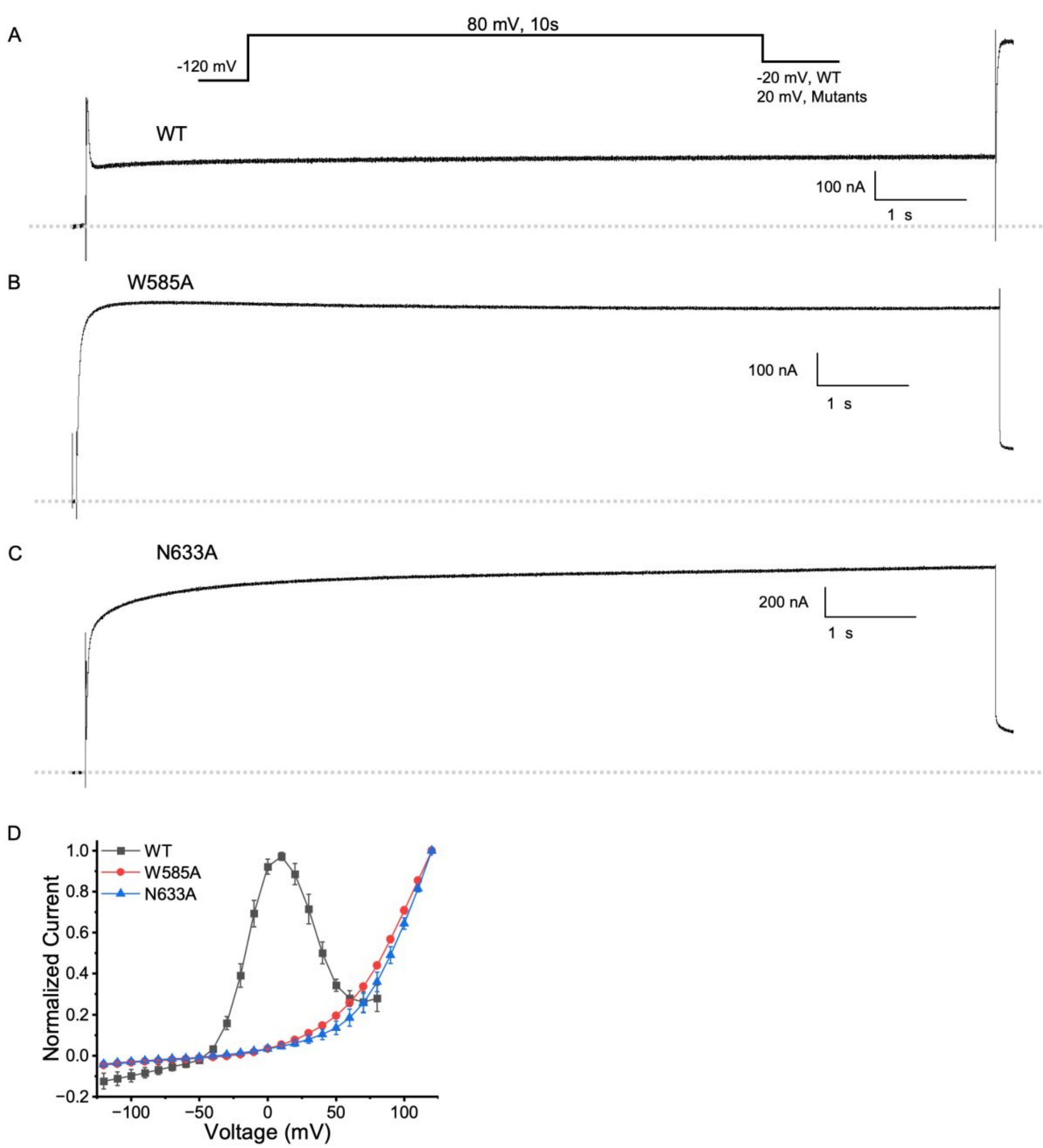

Figure S12. Long depolarizing pulses show that W585A and N633A do not inactivate. (A), (B), and (C) are the representative current traces of WT, W585A, N633A for a 10 s depolarizing voltage pulse 80 mV, respectively. Inset in (A) is the voltage protocol used. The hyperpolarizing returning pulse was -20 mV for WT and 20 mV for the mutants. (D) Current voltage relationship for the currents displayed on Fig. 4D, but in this case not corrected for the leak. Currents were taken from the peak. The leak was corrected by individually fitting using a linear equation the currents in the voltage range where the channels are not open, from -120 to -80 mV. Then, we use the fitted values to correct the hERG currents from the small leak from the oocytes.

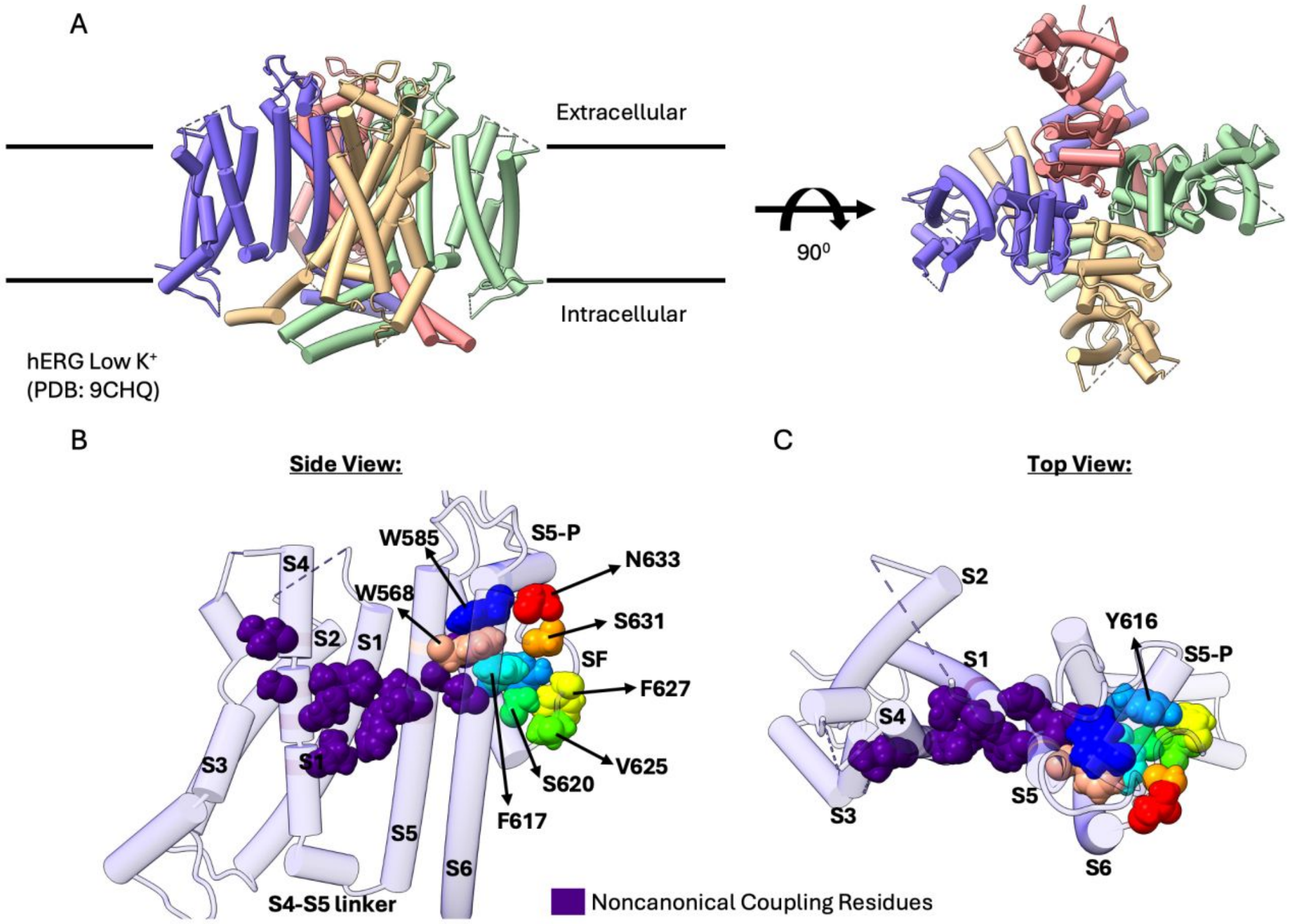

Figure S13: Role of W568 in hERG inactivation. (A) Intramembrane and extracellular views of the inactivated state of hERG (PDB 9CHQ), colored by subunits. (B-C) Intramembrane and top views of single hERG subunit highlighting the residues involved in the noncanonical coupling (T425, P426, L524, A527, L529, L532, H562, W563, A565, A614, and T618 are colored in indigo color, W568, Y616, F617, F627 are shown in the panels) and residues involved in hERG inactivation (W585, S620, V625, F627, S631, N633). W568 is located between F617 and W585, most likely forming a π-π stack interaction. W568 is part of the noncanonical coupling mechanism, connecting the voltage-sensor to the selectivity filter, and it is located between the residues F617 and W585.

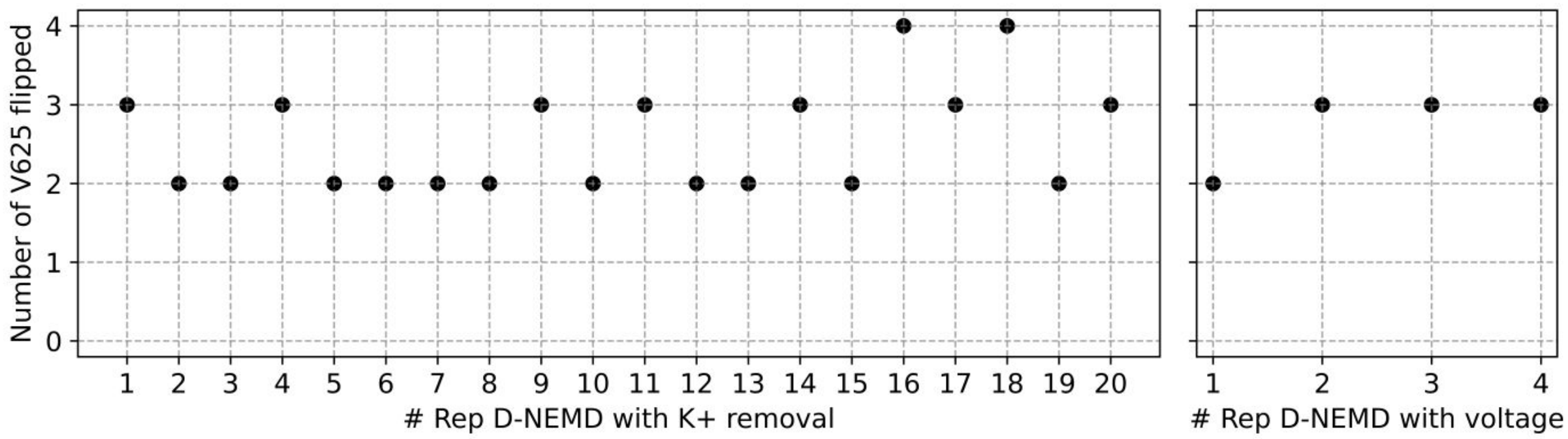

Figure S14: Number of V625 flipped at the end of the D-NEMD simulations.

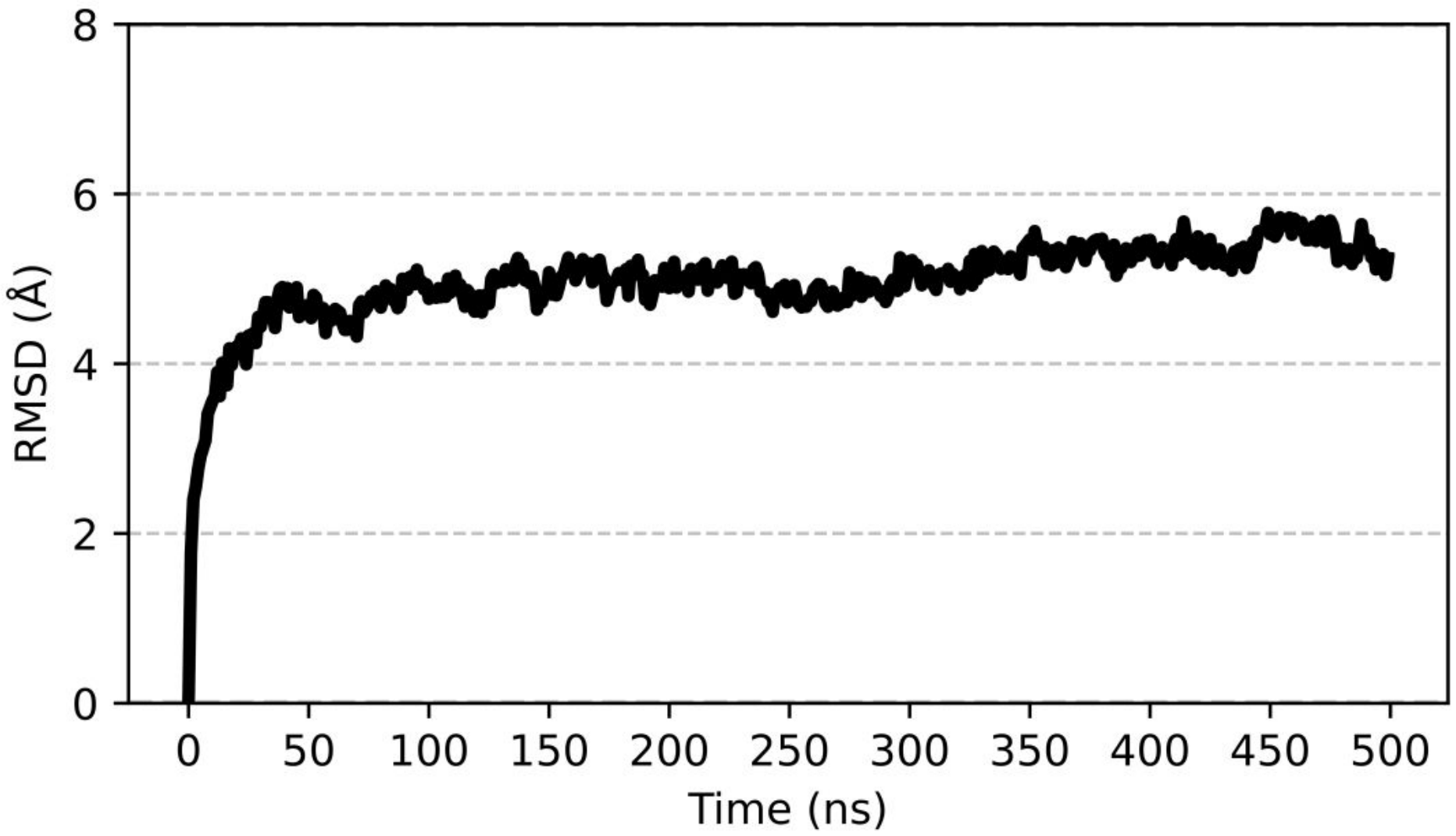

Figure S15. Root Mean Square Displacement (RMSD) profile of the hERG system run at 0 mV with the WKWKW SF configuration. The RMSD was calculated relative to the starting conformation at the beginning of each simulation. A structural superposition between the current and reference conformations was performed using all backbone atoms.

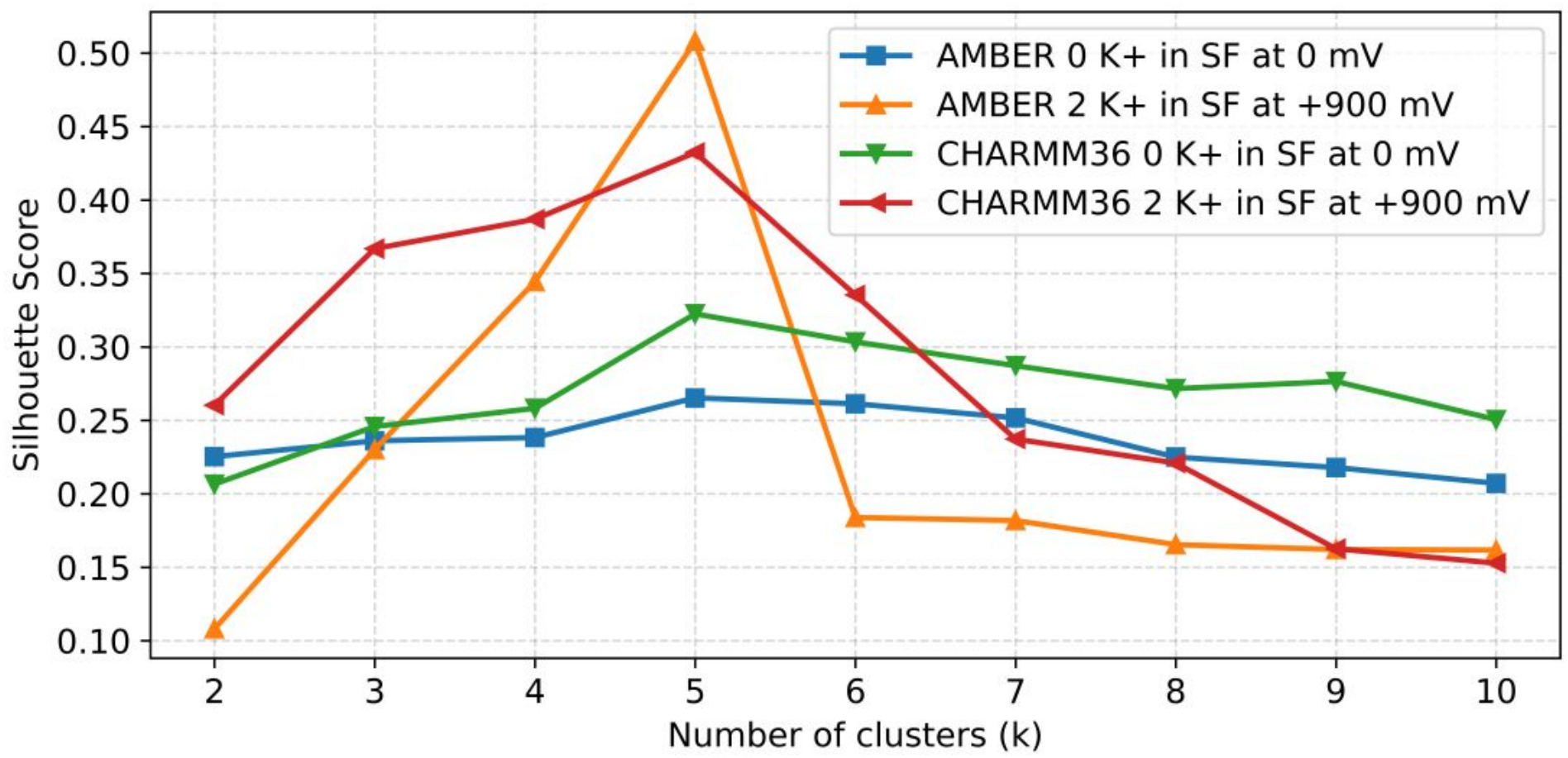

Figure S16. Silhouette scores of the cluster analysis.

Residue	C_α 5 ns	SD	C_α 10 ns	SD	C_α 15 ns	SD	C_α 20 ns	SD	C_α 25 ns	SD
N573	2.22	0.52	3.14	0.98	4.15	1.84	4.54	1.59	5.36	1.40
M574	2.66	0.67	3.53	0.92	4.48	1.62	4.84	1.44	5.78	1.41
E575	2.98	0.63	3.61	0.98	4.64	1.61	5.08	1.59	5.94	1.36
Q576	3.76	0.77	4.41	1.00	5.49	1.86	5.81	1.34	6.59	1.21
P577	4.24	0.82	5.16	0.98	6.24	1.73	6.36	1.41	6.91	1.30
H578	6.87	1.20	6.81	1.40	7.35	1.86	7.61	1.76	7.81	1.53
M579	5.94	1.00	6.23	1.10	6.86	1.94	7.24	1.96	7.36	1.71
D580	5.06	1.00	5.64	1.30	6.64	1.87	6.96	2.04	7.24	1.60
S581	3.13	0.84	4.28	0.79	5.31	1.89	5.79	1.67	6.16	1.34
R582	3.04	0.82	3.97	0.83	5.00	1.75	5.31	1.59	5.96	1.26
I583	2.51	0.68	3.58	1.20	4.84	1.76	5.11	1.51	5.88	1.44
G584	2.43	0.45	3.47	0.86	4.26	1.64	4.56	1.30	5.34	1.17
W585	2.25	0.34	3.23	0.89	4.14	1.71	4.44	1.36	5.14	1.18
L586	2.14	0.33	3.03	0.88	4.06	1.80	4.45	1.31	5.10	1.21
H587	2.28	0.38	3.14	0.93	4.24	1.73	4.54	1.36	5.26	1.21
N588	2.32	0.37	3.23	1.00	4.31	1.74	4.66	1.34	5.37	1.21
L589	2.31	0.35	3.24	0.89	4.13	1.84	4.57	1.37	5.26	1.10
G590	2.54	0.46	3.36	0.80	4.28	1.86	4.67	1.29	5.38	1.14
D591	2.92	0.61	3.64	0.91	4.44	1.83	4.89	1.28	5.39	1.04
Q592	2.93	0.70	3.78	1.00	4.56	1.89	4.91	1.29	5.47	1.13
I593	3.04	0.67	3.72	1.00	4.48	1.94	4.90	1.23	5.60	1.11
G594	3.09	0.77	4.03	1.30	5.07	1.94	5.36	1.34	5.86	1.30
K595	3.48	1.00	4.61	1.40	5.39	2.02	5.51	1.36	6.33	1.30
P596	3.78	1.10	5.25	1.60	5.91	2.07	6.24	1.78	7.04	1.51
Y597	3.24	0.92	5.38	1.50	6.30	1.88	6.59	1.67	7.44	1.54
N598	4.11	1.40	6.77	1.60	7.53	2.06	8.06	2.03	8.96	1.94
S599	4.10	1.30	6.52	1.60	8.05	1.81	8.21	2.00	9.30	1.72
S600	4.60	1.00	6.16	1.30	8.06	1.71	8.10	1.58	8.88	1.62
G601	5.65	1.10	7.34	1.80	8.50	1.93	8.69	1.60	8.97	2.43
L602	4.75	0.77	7.05	1.80	8.05	1.85	8.20	1.89	8.61	2.21
G603	3.64	0.89	5.06	1.50	5.96	1.94	6.01	1.81	6.70	1.82

G604	2.36	0.60	3.60	1.10	4.56	2.18	4.91	1.76	5.76	1.61
P605	2.12	0.55	3.22	1.10	4.24	2.09	4.69	1.51	5.37	1.60
S606	2.45	0.46	3.40	0.94	4.27	1.98	4.55	1.36	5.36	1.30
I607	2.21	0.47	3.20	0.95	4.06	1.97	4.47	1.37	5.24	1.31
K608	1.94	0.40	2.95	0.90	3.78	1.80	4.26	1.38	5.06	1.30
D609	2.09	0.37	2.98	0.87	3.67	1.96	4.13	1.39	5.01	1.20
K610	1.86	0.39	2.86	0.89	3.69	1.90	4.07	1.31	4.93	1.24
Y611	1.72	0.36	2.77	0.90	3.55	1.86	4.03	1.36	4.90	1.22
V612	1.71	0.32	2.79	0.89	3.54	1.84	4.07	1.37	4.84	1.21
T613	1.70	0.35	2.75	0.84	3.46	1.94	4.09	1.36	4.89	1.13
A614	1.64	0.37	2.64	0.86	3.37	1.89	3.94	1.33	4.87	1.10
L615	1.58	0.39	2.66	0.91	3.39	1.86	3.87	1.27	4.71	1.14
Y616	1.54	0.39	2.66	0.89	3.34	1.81	3.84	1.31	4.71	1.11
F617	1.54	0.39	2.52	0.90	3.39	1.84	3.89	1.30	4.76	1.11
T618	1.56	0.41	2.53	0.92	3.30	1.77	3.86	1.26	4.74	1.10
F619	1.47	0.44	2.49	0.94	3.34	1.74	3.84	1.14	4.70	1.00
S620	1.54	0.44	2.64	0.92	3.31	1.79	3.89	1.29	4.78	1.01
S621	1.52	0.45	2.56	0.94	3.34	1.76	3.80	1.24	4.71	1.02
L622	1.50	0.42	2.68	0.89	3.37	1.51	3.87	1.17	4.70	1.03
T623	1.58	0.48	2.57	0.96	3.28	1.50	3.73	1.11	4.58	1.01
S624	1.55	0.54	2.56	0.94	3.26	1.67	3.76	1.21	4.66	1.02
V625	1.61	0.51	2.64	1.00	3.22	1.74	3.86	1.26	4.69	1.00
G626	2.03	0.53	2.86	0.90	3.66	1.63	4.08	1.24	4.74	1.00
F627	1.71	0.61	2.78	0.83	3.51	1.85	4.04	1.31	4.80	1.17
G628	2.28	0.50	3.17	0.77	3.91	1.81	4.47	1.39	5.23	1.36
N629	2.19	0.34	3.03	0.82	3.92	1.88	4.39	1.41	5.04	1.31
V630	2.15	0.36	3.01	0.80	3.76	1.89	4.12	1.30	5.01	1.26
S631	2.64	0.39	3.37	0.77	3.90	1.87	4.33	1.36	5.26	1.34
P632	2.06	0.31	2.86	0.83	3.69	1.84	4.10	1.37	4.99	1.25
N633	2.18	0.35	3.01	0.76	3.88	1.99	4.31	1.46	5.04	1.26
T634	2.19	0.35	3.04	0.84	3.99	1.84	4.46	1.44	5.18	1.30

Table S1. C_α displacement of the amino acid residues after 5, 10, 15, 20 and 25 ns of the K^+ ions removal in the D-NEMD simulations. The values are the mean over the four subunits and the 20 independent D-NEMD simulations ± standard deviation.

Residue	Δx	SD	Δy	SD	Δz	SD
N573	-0.55	0.49	0.27	0.56	-0.04	0.58
M574	-0.65	0.74	-0.13	1.42	1.34	1.33
E575	1.01	1.06	-1.13	0.75	1.73	1.90
Q576	0.89	2.18	0.89	1.07	0.43	2.33
P577	0.11	3.19	0.38	1.60	-0.38	2.17
H578	-0.16	2.72	2.87	2.12	-1.48	3.04
M579	-0.47	2.40	2.37	2.08	-2.74	2.83
D580	0.19	2.69	1.81	2.30	-2.42	1.98
S581	-1.13	2.04	1.04	1.78	-1.06	1.43
R582	-0.94	1.23	1.51	1.26	-0.78	0.88
I583	-1.32	1.38	0.06	0.44	-0.32	0.91
G584	-0.04	0.79	-1.06	0.64	0.48	0.88
W585	0.07	0.67	-1.20	0.57	0.62	0.20
L586	0.18	0.59	-1.02	0.49	0.80	0.18
H587	0.08	0.58	-0.56	0.48	0.72	0.19
N588	-0.07	0.58	-1.30	0.70	0.65	0.33
L589	0.45	0.61	-1.36	0.68	0.50	0.43
G590	0.83	0.15	-1.25	0.65	0.38	0.46
D591	0.84	0.31	-1.69	0.82	0.65	0.65
Q592	0.89	0.27	-1.91	0.91	0.77	0.86
I593	1.18	0.45	-1.85	0.83	0.38	0.52
G594	0.56	0.36	-2.54	1.95	-0.19	0.84
K595	-0.23	1.40	-2.60	1.62	1.01	1.27
P596	-0.45	1.90	-2.88	1.66	1.52	1.75
Y597	-0.86	1.73	-2.02	1.67	-0.88	2.86
N598	-1.10	2.66	-4.95	3.16	1.84	2.75
S599	-3.40	4.73	-3.15	4.48	0.63	2.83
S600	-4.88	4.37	-2.03	4.91	-1.18	3.41
G601	-4.72	5.27	-1.61	4.32	-3.81	3.16
L602	-1.88	4.34	-3.60	2.47	-4.27	2.19
G603	-0.80	2.97	-1.71	0.74	-1.90	0.97
G604	-1.09	1.39	-1.23	1.16	-0.37	0.23
P605	0.63	0.99	-0.48	0.62	-1.07	0.31
S606	1.86	0.62	-0.32	0.36	-0.68	0.53
I607	1.33	0.44	-0.01	0.41	-0.99	0.57
K608	0.87	0.34	0.44	0.15	-0.44	0.48
D609	1.09	0.51	0.06	0.23	0.53	0.32
K610	0.79	0.52	0.33	0.29	0.13	0.33
Y611	0.77	0.44	0.45	0.32	0.09	0.32
V612	0.84	0.44	0.22	0.28	0.15	0.32

T613	0.77	0.45	0.10	0.30	0.45	0.26
A614	0.63	0.40	0.32	0.22	0.33	0.33
L615	0.48	0.36	0.40	0.34	0.35	0.30
Y616	0.41	0.29	0.28	0.29	0.34	0.23
F617	0.34	0.34	0.36	0.31	0.28	0.21
T618	0.24	0.34	0.08	0.40	0.23	0.16
F619	0.30	0.36	-0.13	0.39	0.24	0.20
S620	0.01	0.45	-0.43	0.43	0.05	0.41
S621	-0.23	0.62	-0.13	0.39	0.18	0.34
L622	-0.29	0.56	-0.33	0.53	0.22	0.24
T623	-0.44	0.52	0.10	0.54	0.41	0.38
S624	-0.36	0.41	-0.07	0.52	0.82	0.45
V625	0.02	0.39	-0.23	0.57	1.06	0.50
G626	-0.33	1.08	0.12	0.83	0.68	0.65
F627	0.03	0.82	0.54	0.92	1.03	0.41
G628	0.26	0.42	-0.09	1.01	0.47	0.63
N629	1.22	0.23	0.84	0.64	0.84	0.63
V630	1.25	0.30	1.04	0.57	0.79	0.29
S631	0.96	0.33	1.33	0.55	1.92	0.48
P632	0.95	0.34	1.07	0.39	0.75	0.32
N633	1.43	0.46	0.61	0.60	0.81	0.41
T634	1.10	0.47	1.15	0.57	-0.26	0.28

Table S2. Cartesian coordinates of C_α displacement extracted from the ensemble D-NEMD simulations after 25 ns from the removal of K^+ ions in the SF. Mean values and standard deviation (SD) are reported.

Residue	C_α 30 ns	SD	C_α 60 ns	SD	C_α 90 ns	SD	C_α 120 ns	SD	C_α 150 ns	SD
N573	5.85	0.48	6.53	2.08	6.90	1.50	9.43	0.99	10.64	3.44
M574	6.34	0.61	6.84	2.10	7.31	1.74	9.44	0.92	11.17	3.84
E575	5.99	0.74	7.16	1.93	7.20	1.69	9.26	0.77	11.64	3.65
Q576	6.64	1.30	7.84	1.71	8.23	1.38	9.43	0.74	11.61	4.32
P577	6.73	1.70	9.19	1.69	9.34	1.54	10.82	0.93	12.18	3.81
H578	8.00	1.50	9.60	2.08	10.15	2.07	10.46	0.50	13.06	4.90
M579	7.67	1.20	9.45	1.94	9.86	1.56	10.14	0.67	12.16	4.42
D580	7.66	1.60	8.91	1.96	9.94	1.48	11.36	0.97	12.78	4.17
S581	6.51	1.60	7.86	2.64	8.52	1.64	10.94	1.10	12.91	4.38
R582	6.47	1.60	7.63	2.56	7.61	1.63	9.46	1.30	11.75	4.33
I583	6.13	0.99	7.50	2.75	7.44	1.92	9.50	0.99	11.46	4.02
G584	6.09	1.10	7.54	2.54	7.46	1.81	9.55	0.79	11.51	3.98
W585	5.98	1.10	7.33	2.60	7.28	1.94	9.54	0.86	11.43	3.77
L586	6.07	1.10	7.22	2.63	7.14	1.98	9.61	0.86	11.16	3.99
H587	6.03	1.10	7.40	2.72	7.26	2.25	9.65	0.90	11.17	4.27
N588	6.19	1.00	7.54	2.74	7.30	2.21	9.54	1.10	11.62	4.14
L589	6.26	1.00	7.38	2.63	7.24	2.26	9.67	0.97	11.12	4.19
G590	6.02	0.72	7.39	2.84	7.32	2.28	9.68	0.63	11.18	4.37
D591	6.04	0.63	7.46	3.08	7.31	2.34	9.66	0.81	11.51	4.30
Q592	6.41	0.83	7.41	2.79	7.47	2.26	9.71	0.96	11.56	4.20
I593	6.66	0.66	7.44	2.84	7.49	2.24	9.73	0.80	11.56	4.27
G594	6.40	0.64	7.63	3.36	7.84	2.56	9.50	1.04	11.82	4.41
K595	7.44	0.33	8.21	3.13	8.01	1.58	10.61	1.49	12.69	3.39
P596	8.39	0.34	9.40	1.94	9.30	0.53	11.81	2.28	13.26	1.57
Y597	7.91	0.38	10.16	1.29	10.12	1.66	12.46	2.80	14.45	0.79
N598	9.99	1.20	11.69	0.85	12.80	2.24	14.12	3.11	16.35	1.10
S599	9.89	1.30	10.13	1.47	12.69	2.48	14.04	3.20	16.27	1.24
S600	9.34	0.94	10.82	1.76	11.45	2.43	13.32	2.91	16.15	0.56
G601	8.84	1.00	10.46	1.91	11.01	2.24	12.84	2.09	14.15	2.02
L602	8.16	0.59	10.00	2.13	9.86	2.25	10.16	2.40	13.74	2.57
G603	6.78	0.17	8.43	2.25	8.44	2.03	9.51	1.88	11.61	2.85

G604	6.29	0.45	7.84	2.58	7.69	2.01	9.63	1.46	11.64	3.40
P605	5.97	0.85	7.06	2.44	7.25	2.04	9.36	1.32	11.74	3.94
S606	5.83	0.94	7.08	2.35	7.14	1.96	9.44	1.35	11.89	3.88
I607	5.71	1.00	6.91	2.36	7.03	1.74	9.34	1.37	11.80	3.76
K608	5.70	1.00	6.80	2.24	6.86	1.88	9.46	1.33	11.46	3.50
D609	5.93	0.89	6.94	2.22	6.84	1.95	9.53	1.38	11.30	3.54
K610	5.77	0.96	6.93	2.27	6.87	1.87	9.31	1.29	11.49	3.55
Y611	5.76	0.97	6.73	2.14	6.70	1.78	9.47	1.23	11.12	3.39
V612	5.68	0.96	6.63	2.03	6.76	1.86	9.38	1.31	10.32	3.45
T613	5.67	0.94	6.64	2.04	6.75	1.73	9.41	1.28	11.56	3.40
A614	5.61	0.94	6.63	2.08	6.76	1.64	9.33	1.24	10.51	3.30
L615	5.64	0.92	6.50	1.94	6.61	1.62	9.36	1.31	10.69	3.17
Y616	5.59	0.91	6.50	2.04	6.64	1.64	9.36	1.36	10.41	3.21
F617	5.51	0.93	6.43	2.05	6.65	1.63	9.24	1.34	10.68	3.38
T618	5.54	0.87	6.36	1.91	6.52	1.51	9.26	1.59	10.74	3.10
F619	5.56	0.94	6.44	1.81	6.63	1.55	9.34	1.48	10.46	3.11
S620	5.54	0.93	6.47	1.82	6.64	1.49	9.34	1.46	10.89	3.22
S621	5.44	0.87	6.39	1.81	6.52	1.44	9.23	1.57	10.65	3.18
L622	5.53	0.78	6.27	1.80	6.41	1.33	9.16	1.62	10.47	2.96
T623	5.56	0.83	6.36	1.71	6.44	1.51	9.24	1.55	10.12	2.94
S624	5.64	0.85	6.43	1.70	6.33	1.54	9.43	1.40	10.68	2.95
V625	5.61	0.98	6.44	1.85	6.30	1.61	9.30	1.30	10.61	3.04
G626	5.81	1.20	6.88	1.88	6.70	1.48	9.44	1.70	10.47	3.16
F627	5.63	1.10	6.56	1.70	6.54	1.59	9.36	1.40	11.65	3.14
G628	5.90	1.20	7.08	1.72	6.82	1.77	9.84	1.44	11.14	3.07
N629	6.01	1.10	7.27	2.21	6.91	2.23	9.83	0.65	11.94	3.33
V630	6.14	0.94	7.11	2.10	7.04	2.08	9.83	0.80	11.47	3.44
S631	6.76	0.70	7.60	2.00	7.18	1.97	10.04	0.80	11.69	3.74
P632	5.83	0.80	6.91	2.06	6.86	1.63	9.55	0.90	11.41	3.68
N633	5.85	0.77	7.01	2.14	6.91	1.95	9.54	0.97	11.47	3.77
T634	5.63	0.86	6.92	2.14	7.00	1.92	9.33	1.10	11.95	3.74

Table S3. C_α displacement of the amino acid residues after 30, 60, 90, 120 and 150 ns the electric field perturbation in the D-NEMD simulations. The values are the mean over the four subunits and the 4 independent D-NEMD simulations ± standard deviation.

Residue	Δx	SD	Δy	SD	Δz	SD
N573	1.49	0.58	2.07	1.01	-0.44	0.63
M574	0.29	0.39	3.09	1.19	-0.58	0.78
E575	1.63	1.75	1.46	1.93	0.37	1.22
Q576	3.03	2.90	1.86	1.73	-0.54	0.92
P577	0.52	2.97	-0.34	2.96	-1.40	1.51
H578	-0.52	2.70	2.73	4.63	-3.30	1.73
M579	0.57	3.15	2.79	3.04	-3.83	0.17
D580	2.28	1.20	3.39	2.17	-3.72	0.69
S581	2.04	1.48	1.86	1.69	-2.59	1.60
R582	1.81	2.57	2.14	0.61	-1.30	0.26
I583	-0.37	1.61	0.32	1.95	-0.11	1.08
G584	0.41	1.21	-0.50	1.23	0.63	0.47
W585	0.62	0.98	-0.53	1.30	0.47	0.36
L586	0.72	0.75	-0.28	1.25	0.70	0.44
H587	0.63	1.06	0.19	1.29	0.56	0.44
N588	0.52	1.03	-0.54	1.27	0.56	0.72
L589	1.07	0.74	-0.64	1.23	0.53	0.63
G590	1.41	1.03	-0.33	1.36	0.43	0.40
D591	1.49	1.26	-0.64	1.43	0.78	0.81
Q592	1.55	0.89	-1.04	1.23	1.03	1.28
I593	1.83	0.97	-1.00	1.26	0.88	1.00
G594	1.50	2.17	-1.90	0.81	-0.01	1.48
K595	0.82	1.65	-1.71	0.83	1.12	2.07
P596	1.04	2.71	-3.06	1.82	1.49	1.22
Y597	0.26	2.38	-3.23	2.89	-0.84	2.19
N598	0.81	3.09	-7.60	4.53	1.59	2.07
S599	-0.54	2.91	-6.86	6.56	0.77	3.19
S600	-1.91	1.12	-6.20	6.17	-0.17	3.59
G601	-2.63	0.75	-4.46	4.82	-2.85	1.16
L602	0.30	1.86	-6.26	3.02	-4.42	1.36
G603	0.55	3.32	-3.61	1.18	-2.54	1.15
G604	1.19	2.90	-1.79	1.21	-0.24	0.37
P605	2.02	2.01	-0.40	0.67	-0.19	0.69
S606	2.24	0.68	0.04	0.64	-0.05	0.48
I607	1.69	0.56	0.66	0.84	-0.26	0.52
K608	1.24	0.74	0.75	0.85	0.29	0.33
D609	1.38	0.52	0.46	0.69	1.06	0.36
K610	1.01	0.41	0.78	0.78	0.59	0.21
Y611	1.00	0.56	0.55	0.63	0.61	0.24
V612	1.09	0.61	0.20	0.61	0.54	0.34

T613	0.90	0.51	0.18	0.62	0.51	0.26
A614	0.78	0.42	0.27	0.57	0.44	0.19
L615	0.67	0.45	0.12	0.42	0.54	0.22
Y616	0.55	0.43	0.03	0.57	0.43	0.20
F617	0.33	0.42	0.17	0.53	0.40	0.34
T618	0.29	0.46	-0.07	0.41	0.33	0.44
F619	0.17	0.53	-0.12	0.39	0.55	0.26
S620	0.23	0.32	-0.09	0.30	0.49	0.36
S621	0.10	0.32	-0.04	0.40	0.14	0.19
L622	-0.07	0.38	-0.61	0.52	0.29	0.20
T623	-0.22	0.40	-0.34	0.34	0.62	0.28
S624	-0.20	0.17	-0.49	0.20	0.96	0.37
V625	-0.19	0.26	-0.55	0.27	0.89	0.28
G626	-0.27	0.13	0.23	0.11	0.51	0.28
F627	-0.06	0.32	0.42	0.56	0.73	0.13
G628	0.36	0.60	-0.29	2.11	0.14	0.78
N629	1.62	0.33	1.09	0.69	0.30	0.64
V630	1.72	0.32	1.21	0.60	0.63	0.41
S631	1.15	0.51	1.37	0.55	1.61	0.74
P632	1.20	0.58	1.01	0.57	0.51	0.32
N633	1.33	1.00	1.29	0.55	0.22	0.39
T634	1.27	0.92	1.48	0.66	-0.34	0.66

Table S4. Cartesian coordinates of C_α displacement extracted from the ensemble D-NEMD simulations after 150 ns from the application of the +900 mV electric field. Mean values and standard deviation (SD) are reported.

Force Field	Initial SF Configuration	Voltage	Replicas and Simulation Time
AMBER	WKWKW	0 mV	1 replica of 500 ns
AMBER	WWWKK	+500 mV	1 replica of 500 ns
AMBER	WWWKK	+700 mV	1 replica of 500 ns
AMBER	WWWKK	0 mV	3 replicas of 300 ns + 1 replica of 500 ns
AMBER	WWWWW	0 mV	1 replica of 150 ns + 2 replicas of 750 ns
AMBER	WWWKK	+900 mV	2 replicas of 100 ns + 1 replica of 300 ns
CHARMM36	WWWKK	0 mV	1 replica of 300 ns + 1 replica of 500 ns
CHARMM36	WWWWW	0 mV	1 replica of 300 ns + 1 replica of 400 ns
CHARMM36	WWWKK	+900 mV	3 replicas of 250 ns + 1 replica of 150 ns

Table S5. Summary of the equilibrium MD simulations considered in this study with the relevant features.

Movie S1. Structural representation of the cartesian coordinates of C_α displacement extracted from the ensemble D-NEMD simulations after 25 ns from the removal of K+ ions in the SF. Side view of the protein where only the pore domain (from helix S5 to helix S6) is shown. The S5-P helix is in red, P-helix in blue and the Selectivity Filter in orange.

Movie S2. Structural representation of the cartesian coordinates of C_α displacement extracted from the ensemble D-NEMD simulations after 25 ns from the removal of K+ ions in the SF. Top view of the protein where only the pore domain (from helix S5 to helix S6) is shown. The S5-P helix is in red, P-helix in blue and the Selectivity Filter in orange.

Movie S3. Formation and breaking of intra-subunit contacts between residues on the SF and P-helix in the ensemble of 25 ns D-NEMD simulations where the removal of the ions was used as a perturbation.

Movie S4. Formation and breaking of intra-subunit contacts between residues on the SF and P-helix in the ensemble of 150 ns D-NEMD simulations where the high voltage at +900 mV was used as a perturbation.
